# Bibliometric study and visualization of myocardial infarction combined with heart failure, 1993–2023

**DOI:** 10.3389/fcvm.2025.1555748

**Published:** 2025-08-08

**Authors:** Kexin Wang, Shaoqiang Zhang, Mingdan Zhu

**Affiliations:** ^1^ Graduate School of Tianjin University of Traditional Chinese Medicine, Tianjin, China; ^2^ Department of Cardiovascular Medicine, Second Affiliated Hospital of Tianjin University of Traditional Chinese Medicine, Tianjin, China

**Keywords:** myocardial infarction, heart failure, bibliometrics, CiteSpace, VOSviewer

## Abstract

**Background:**

The incidence of myocardial infarction has been rapidly increasing in recent years, making it one of the most common cardiovascular disorders. Due to the intricate interactions between large-vessel occlusions, microvascular dysfunction, ventricular remodeling, inflammation, and neurohormonal activation, patients who experience myocardial infarction are more likely to develop heart failure. Even though myocardial infarction and heart failure have been studied extensively, a thorough bibliometric analysis has not yet been carried out. The purpose of this study is to use bibliometric analysis to examine the trends in myocardial infarction linked to heart failure during the previous 30 years.

**Methods:**

From 1993 to 2023, this study methodically retrieved original publications from the Web of Science Core Collection (WoSCC) about myocardial infarction and heart failure. We identified research trends and hotspots in the subject by extracting and analyzing data on countries/regions, institutions, authors, journals, keywords, and references related to the issue using tools like CiteSpace and VOSviewer.

**Results:**

Over the past 30 years, there has been a consistent increase in the number of published articles about myocardial infarction and heart failure, reaching a peak in 2022. The United States and China have a significant advantage in publication volume, each exceeding 200 articles. Brigham and Women's Hospital has published the most articles, totaling 49. In addition to publishing the most papers, the journal Circulation also had the biggest influence. The top five keywords include heart failure, myocardial infarction, mortality, acute myocardial infarction, and cardiovascular disease. In recent years, the outbreak words that have remained in the spotlight are management, association, risk, percutaneous coronary intervention, and guidelines.

**Conclusion:**

Over the past 5 years, the increasing incidence of myocardial infarction accompanied by heart failure has garnered significant attention in research, leading to a substantial growth in related literature. The main goal of current research is to clarify the processes through which myocardial infarction causes heart failure; predictions and biomarkers are important areas of study. Future research is likely to concentrate on screening methods and treatment strategies.

## 1 Introduction

The clinical syndrome known as heart failure (HF) is defined by abnormal structural or functional alterations in the heart due to a variety of causes. Myocardial infarction (MI) is caused by atherosclerotic lesions in the coronary arteries that reduce blood flow, resulting in severe ischemia in the affected myocardium and subsequent necrosis of a portion of the heart muscle. These changes result in impaired ventricular systolic and/or diastolic function, increased cardiac load, decreased cardiac output, elevated pressures in the systemic or pulmonary circulation, and inadequate tissue perfusion. Heart failure, which is linked to a poor prognosis and high mortality, is the most severe and fatal stage of cardiovascular illness.

According to studies, heart failure affects 14%–36% of hospitalized patients with acute myocardial infarction (AMI) and is the primary cause of death among these patients (1). Cardiovascular disease (CVD) remains the leading cause of death in both China and the United States, with AMI and subsequent heart failure as primary contributors (2, 3). Post-infarction, coronary occlusion induces ischemic necrosis of cardiomyocytes, leading to fibrotic scar formation, diminished contractile capacity, and progressive ventricular remodeling—manifested by myocardial fibrosis and hypertrophy—ultimately resulting in heart failure.

The field of bibliometrics uses statistical and mathematical techniques to perform quantitative analysis on scholarly publications. Its primary objective is to uncover patterns in academic research activities, trends in disciplinary development, and the dissemination laws of knowledge by analyzing literature and associated data (such as publications, citations, keywords, etc.). It serves as an impartial and dependable method for assessing the influence and significance of research results. Visualization tools utilize data mining techniques to uncover useful insights and present them from various perspectives. Using the extracted data, knowledge graphs can be developed to assess academic output, identify major research hotspots, and forecast future developments. Over the last 30 years, a significant amount of research has been published on myocardial infarction combined with heart failure, making manual information extraction progressively difficult. Therefore, bibliometric software is essential for systematically summarizing research findings in this domain. CiteSpace and VOSviewer are the most often used bibliometric tools among a variety of software programs. Through three-dimensional visualizations such as Network Visualization, Overlay Visualization, and Density Visualization, VOSviewer makes it possible to visualize networks of collaboration across nations, organizations, and authors. CiteSpace is a data analysis and visualization tool that focuses on time aspects and is founded on set theory. By analyzing time slices via timelines, CiteSpace delves deeper into identifying trends within the field. Furthermore, co-citation analysis and burst detection make it easier to find groundbreaking research and new keywords, giving a thorough picture of the development of the area.

Despite the availability of bibliometric analyses on heart failure, limited studies have focused on myocardial infarction combined with heart failure. This review compiles a total of 1,095 papers published between January 1993 and December 2023 for a comprehensive bibliometric analysis of myocardial infarction in conjunction with heart failure. This review is organized as follows: The data sources and analytical techniques are described in Section 2; the annual publication trends, contributing nations, authors, institutions, and important research topics are covered in detail in Section 3; and the results are compiled in Section 4, which also addresses the state of the field today and its future directions.

## 2 Methods

### 2.1 Data source and search strategy

**Figure 1:**
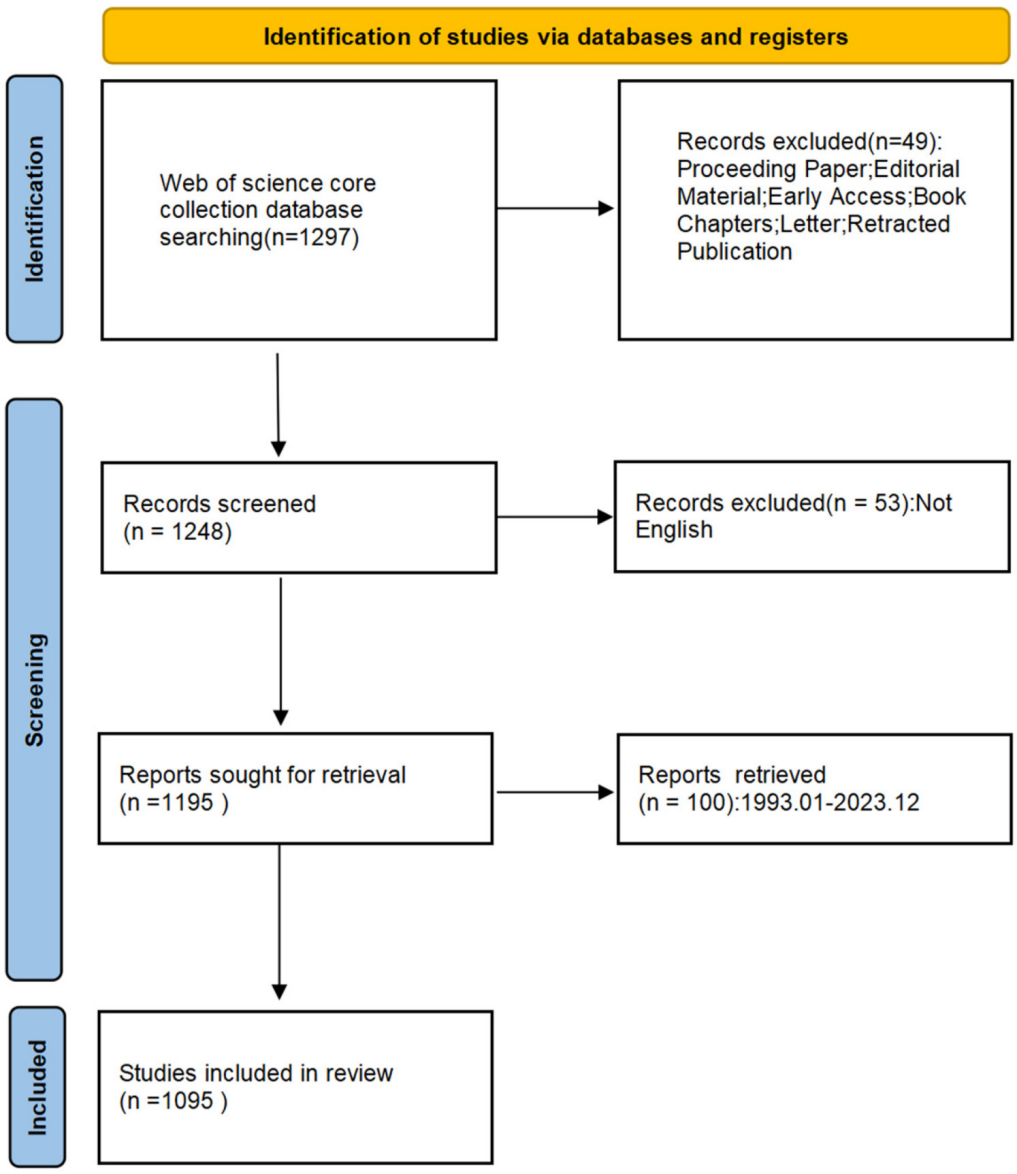
Flowchart of the inclusion and exclusion criteria.

The Web of Science Core Collection (WoSCC) has been chosen as the data source for this investigation. The most prestigious core academic publications in engineering technology, biomedicine, and the natural sciences are all included in WoSCC, the greatest comprehensive academic resource database. By utilizing WoSCC's sophisticated search features, researchers can quickly obtain important scientific data and develop a comprehensive grasp of the subject. The search strategy of this study is “TI = (bibliometrics OR CiteSpace OR VOSviewer) AND TI = (‘heart failure’ OR ‘cardiac failure’ OR ‘heart decompensation’ OR ‘decompensation, heart’ OR ‘myocardial failure’) AND AB = (‘heart failure’ OR ‘cardiac failure’ OR ‘heart decompensation’ OR ‘decompensation, heart’ OR ‘myocardial failure’) AND TI = (‘myocardial infarction’ OR ‘Infarction, Myocardial’ OR ‘Infarctions, Myocardial’ OR ‘Myocardial Infarctions’ OR ‘Heart Attack’ OR ‘Heart Attacks’ OR ‘Myocardial Infarct’ OR ‘Infarct, Myocardial’ OR ‘Infarcts, Myocardial’ OR ‘Myocardial Infarcts’ OR ‘Cardiovascular Stroke’ OR ‘Cardiovascular Strokes’ OR ‘Stroke, Cardiovascular’ OR ‘Strokes, Cardiovascular’) AND AB = (‘myocardial infarction’ OR ‘Infarction, Myocardial’ OR ‘Infarctions, Myocardial’ OR ‘Myocardial Infarctions’ OR ‘Heart Attack’ OR ‘Heart Attacks’ OR ‘Myocardial Infarct’ OR ‘Infarct, Myocardial’ OR ‘Infarcts, Myocardial’ OR ‘Myocardial Infarcts’ OR ‘Cardiovascular Stroke’ OR ‘Cardiovascular Strokes’ OR ‘Stroke, Cardiovascular’ OR ‘Strokes, Cardiovascular’)”. Publication dates ranged from January 1993 to December 2023. Only English-language publications of reviews and articles were included in the search. After 1,095 papers were recovered, the records were exported in tab-delimited file formats and plain text (Figure 1). To minimize data bias, all papers were exported on September 1, 2024.

### 2.2 Data analysis and visualization methods

CiteSpace 6.3, VOSviewer 1.6.20, the Bibliometrix (R package), and RStudio 4.3.2 were the main bibliometric analytic tools used for the bibliometric and visual knowledge graph analysis of authors, countries, institutions, journals, co-cited references, and keyword bursts. Microsoft Office Excel 2019 was utilized for data management and table creation.

## 3 Results

### 3.1 Trend analysis of the number of articles published

**Figure 2:**
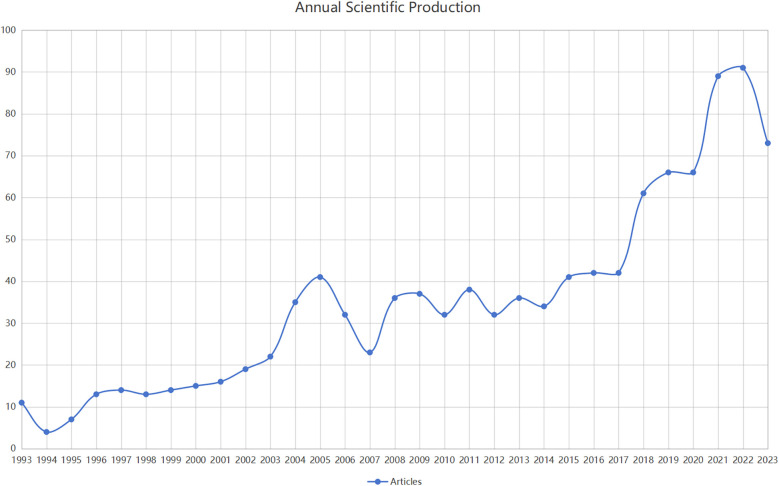
The distribution trend from January 1993 to December 2023.

From January 1993 to December 2023, WoSCC received a total of 1,297 papers on myocardial infarction combined with heart failure, of which 1,095 articles met the screening criteria. The number of publications per year exhibited a generally increasing trend, as illustrated in Figure 2. Since 2008, the annual publication count has consistently exceeded 30. Notably, there was a minor surge in publications from 2003 to 2005, peaking in 2005, followed by a more substantial increase from 2017 to 2022, with a peak of 91 articles in 2022. This trend indicates growing interest and attention in this field.

### 3.2 Cooperation network

#### 3.2.1 Country cooperation networks

From January 1993 to December 2023, 74 countries published articles on myocardial infarction combined with heart failure; the United States (USA) was the most productive and influential country, contributing 385 relevant papers that received a total of 26,213 citations, with an average of 68.09 citations per article. Based on the number of publications per country, we were able to identify influential countries in the field and map the network of national collaborations. There is a significant disparity between the USA and other countries in terms of publication output, indicating a pronounced top-tier effect. Collectively, the top 10 countries accounted for the majority of the publications (Table 1).

**Table 1:** The ten countries with the highest number of published articles.

ID	Country	Documents	Percentage	Citations	Total link strength	Average citation per article
1	Usa	385	22.81%	26,213	419	68.09
2	China	216	12.80%	2,969	36	13.75
3	Canada	92	5.45%	7,946	181	86.37
4	Japan	78	4.62%	2,456	17	31.49
5	England	77	4.56%	4,778	152	62.05
6	France	69	4.09%	2,915	196	42.25
7	Denmark	68	4.03%	6,753	145	99.31
8	Italy	62	3.67%	7,386	189	119.13
9	Germany	58	3.44%	5,014	137	86.45
10	Sweden	56	3.32%	5,867	128	104.77

**Figure 3:**
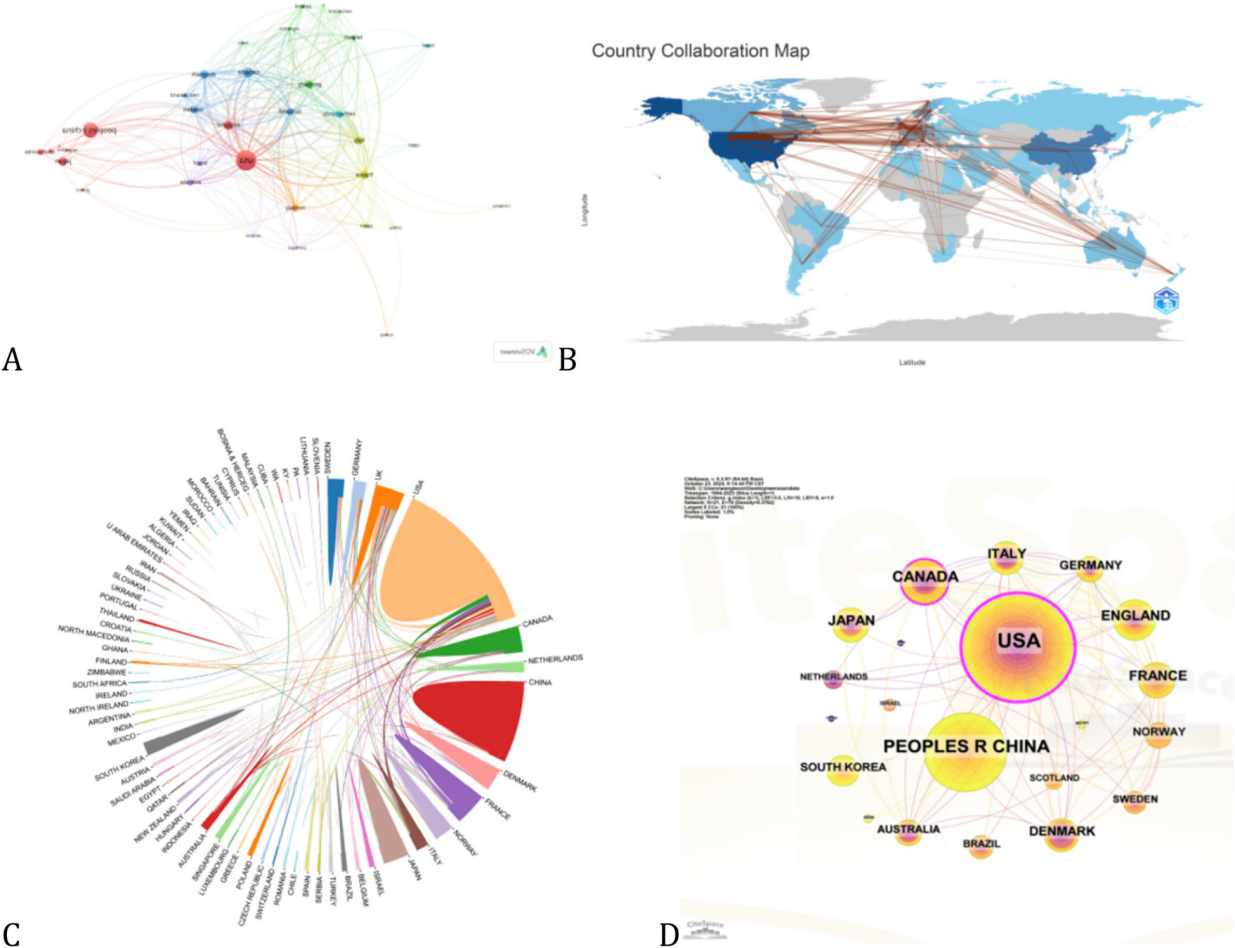
Country cooperation network maps, including **(A–D)**. **(A)** A Country Collaboration Network Map shows research collaboration between countries. Nodes (circles) represent countries, with larger nodes indicating more active participation in collaborations. Edges (lines) connect countries that have co-authored papers or worked together on research projects, with thicker lines indicating stronger or more frequent collaborations. Colors distinguish collaboration clusters, with each color representing a group of closely connected countries. **(B)** The World Collaboration Map visually represents international partnerships based on geographic location. Connecting lines indicate collaboration between countries—thicker lines reflect more frequent cooperation. Darker country colors signify higher levels of collaborative activity, such as joint publications or research engagement. **(C)** The Chord Diagram visually represents bilateral collaboration between countries. Arcs along the outer circle denote individual countries, while connecting chords indicate partnerships—thicker chords reflect stronger ties. Colors distinguish countries and highlight major collaborations. **(D)** The Collaboration Intensity Network Map illustrates global collaboration strength and structure, highlighting leading countries. Nodes represent countries, with size and text weight indicating total collaboration intensity. Thicker connecting lines show stronger bilateral ties, while color and glow effects emphasize key players.

International collaboration is crucial for advancing this field. Based on the co-occurrence network generated by VOSviewer, the United States, China, Canada, Japan, and the United Kingdom have played key roles. With a threshold of six publications, we created a network graph illustrating national cooperation (Figure 3). In this graph, the size or color of the nodes represents publication volume, and the lines connecting the countries show their collaborations. The thickness and number of these lines reflect the strength of their partnerships. The United States, with a link strength of 419, is central to the network, highlighting its dominant role and influence. The top three countries with the most significant ties to the United States are Canada, Italy, and France. Centrality is used to assess the importance of each country within the international collaboration network. A higher centrality value indicates a more critical role in connecting different countries and facilitating knowledge flow across the entire network. According to the calculation (Figure 3D), the United States has the highest centrality (0.46), suggesting it serves as a key bridge in global research collaboration. France and the United Kingdom also exhibit relatively high centrality values (0.38 and 0.27, respectively), reflecting their significant influence and integrative capacity within the network. These findings highlight the prominent intermediary roles played by a few key countries in shaping the global research landscape.

#### 3.2.2 Author collaboration network

The criterion for core authorship can be expressed as follows: *m* ≈ 0.749 × (*n*_max)^0.5^, where *n*_max is the number of papers published by the most prolific author and *m* is the minimum number of papers required for an author to be considered part of the core group. Based on Price's Law, there were 6,291 authors who published papers on myocardial infarction combined with heart failure between January 1993 and December 2023. Authors who have published more than four papers in this field are categorized as core authors, and 85 authors, or 1.35% of the total number of authors (85/6,291).

**Figure 4:**
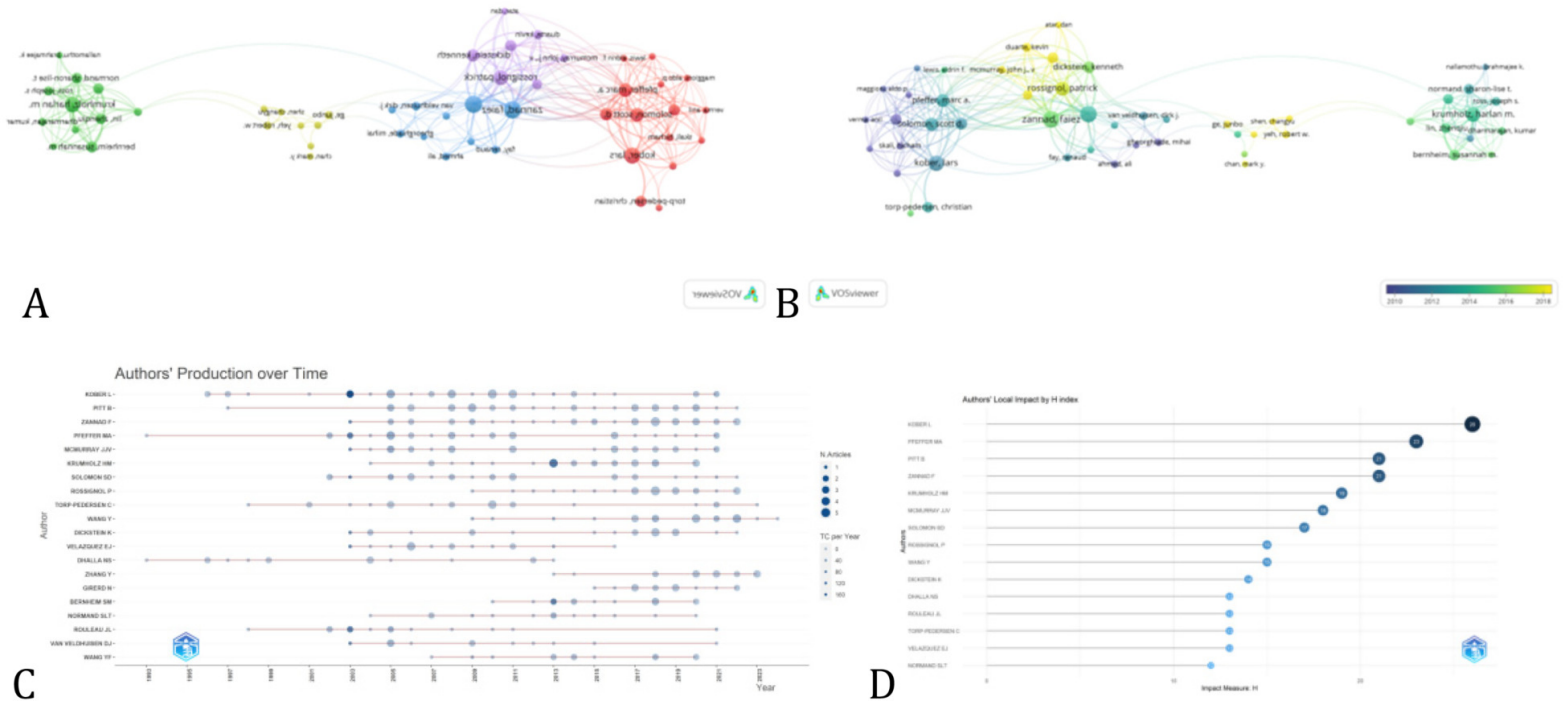
The visualization knowledge maps of author, including **(A)** network visualization, **(B)** overlay visualization, and **(C)** authors’ production over time and **(D)** authors’ local impact by H index.

Four American scholars are among the top 10 most prolific authors (Table 2), highlighting the important role of American researchers in this field. Professor Faiez Zannad of the University of Lorraine wrote 33 papers, with a total link strength of 318 with other researchers. He was the most influential author, with 1,316 total citations and an average citation frequency of 39.88 per paper. Assessing the efficacy of eplerenone in treating patients with myocardial infarction and heart failure is the main focus of Prof. Zannad's research. Figure 4 shows a collaborative network diagram of authors with more than four publications, divided into five clusters overall, with Prof. Faiez Zannad at the center, working closely with 25 co-authors, including Patrick Rossignol and Bertram Pitt as his closest collaborators. Figure 4 highlights that the research group led by Prof. Patrick Rossignol has extensively studied the effects of spironolactone and other inhibitors of the renin-angiotensin-aldosterone system on heart failure patients over the past decade. Additionally, Profs. Harlan M. Krumholz, Lars Kober, and John J.V. McMurray have high H-indexes and average citation frequencies, indicating their leadership in the field.

**Table 2:** The ten authors with the highest number of published articles.

ID	Author	Documents	Countrys	Citations	Total link strength	Average citation per article
1	Zannad, Faiez	33	France	1,316	318	39.88
2	Pitt, Bertram	32	USA	1,283	255	40.09
3	Kober, Lars	27	Denmark	1,494	275	55.33
4	Krumholz, Harlan M.	25	USA	2,690	209	107.60
5	Rossignol, Patrick	23	France	892	221	38.78
6	Pfeffer, Marc A.	21	USA	1,095	260	52.14
7	Solomon, Scott D.	20	USA	1,030	230	51.50
8	Dickstein, Kenneth	17	Norway	585	159	34.41
9	Mcmurray, John j. V.	16	UK	955	198	59.69
10	Torp-Pedersen, Christian	14	Denmark	288	102	20.57

#### 3.2.3 Institutional cooperation network

**Figure 5:**
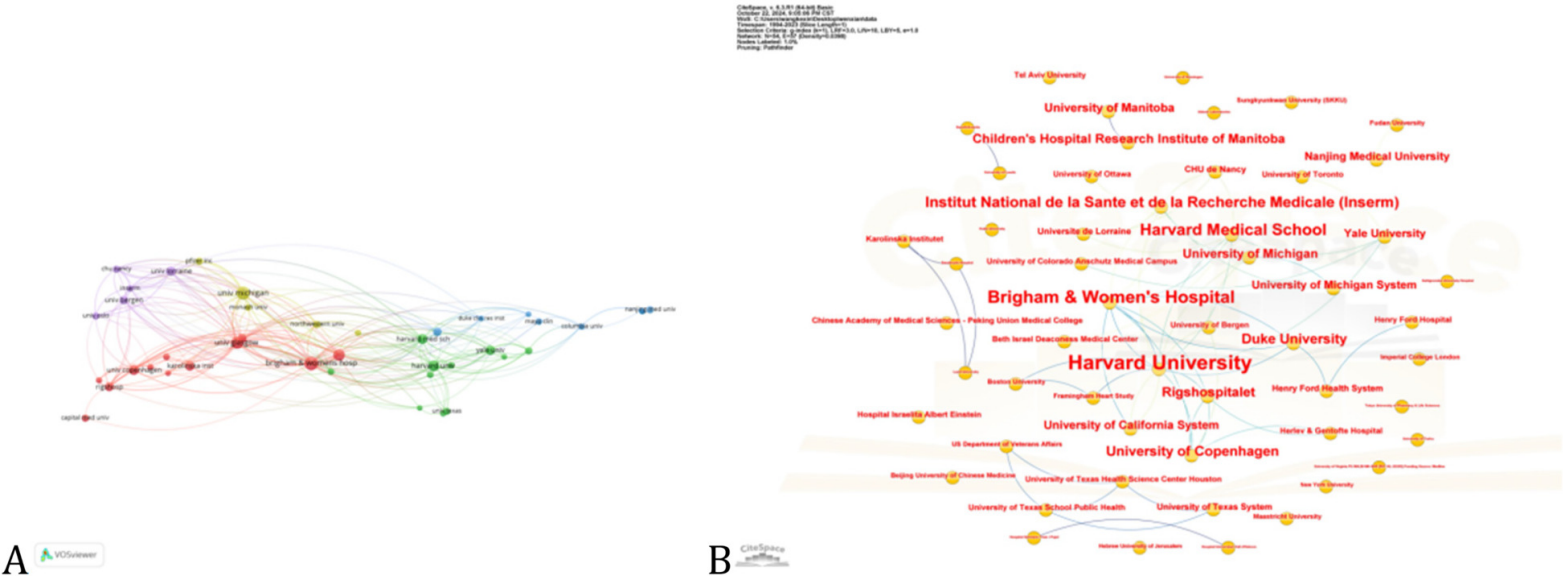
Diagram of the institutional cooperation network. **(A)** The Institution Collaboration Network Map visualizes research partnerships between institutions. Nodes represent institutions, with larger nodes indicating higher collaboration activity. Edges connect co-authoring institutions, with thicker lines signifying stronger ties. Colors highlight clusters of closely connected institutions. **(B)** This image shows a collaboration network of research institutions, where each node represents an institution and lines indicate partnerships such as co-authored papers or joint projects. Larger, bolder nodes—like Harvard University and Brigham & Women's Hospital—highlight central institutions with high levels of collaboration.

Between January 1993 and December 2023, a total of 1,709 institutions were involved in research related to myocardial infarction combined with heart failure. Among them, Brigham and Women's Hospital stood out with 49 publications, showcasing its notable impact in this area. Five of the top 10 institutions (Table 3) were based in the United States, underscoring the country's leading role in medical research. Figure 5 depicts the collaborative network of the 43 leading contributing institutions. Both the University of Glasgow and Brigham and Women's Hospital have link strengths of 309 and 304, respectively, indicating their strong collaborative ties and prominent positions within the field. Notably, Duke University has the strongest links to both Brigham and Women's Hospital and the University of Glasgow, having published numerous high-quality articles. This suggests that institutional collaborations have significantly advanced the field. Furthermore, Brigham and Women's Hospital, the University of Glasgow, and Duke University are all categorized in the red group, indicating that institutions in this group hold greater influence.

**Table 3:** Top ten organizations in terms of number of articles published.

ID	Organization	Documents	Countrys	Citations	Total link strength	Average citation per article
1	Brigham & Womens Hosp	49	USA	4,717	304	96.27
2	Univ Michigan	44	USA	1,851	232	42.07
3	Univ Glasgow	40	UK	3,879	309	96.98
4	Duke Univ	37	USA	4,742	237	128.16
5	Harvard Univ	34	USA	4,696	131	138.12
6	Harvard Med Sch	27	USA	817	166	30.26
7	Univ Copenhagen	26	Denmark	680	114	26.15
8	Karolinska Inst	25	Sweden	1,114	107	44.56
9	Univ Lorraine	25	France	676	213	27.04
10	Univ Manitoba	25	Canada	1,222	27	48.88

### 3.3 Journal analysis

Table 4 lists the 10 journals with the highest publication frequency. Among the 393 journals, the American Journal of Cardiology (IF = 2.3, Q2) ranks first in the number of publications with 63 articles and an average of 32.44 citations per article. The Journal of the American Heart Association (IF = 5.0, Q1) has the highest citation rate with 250.19 citations per article. The European Heart Journal (IF = 37.6, Q1) not only leads in citation frequency but also boasts the highest impact factor, making it one of the most influential journals in cardiovascular research. Circulation (IF = 35.5, Q1) demonstrates a higher impact factor, citation frequency, and H-index relative to other journals. These journals are particularly attractive to researchers due to their high impact factors and substantial contributions to myocardial infarction combined with heart failure.

**Table 4:** The ten journals with the highest number of published articles.

ID	Source	Documents	IF	Citations	Total link strength	Average citation per article
1	American Journal of Cardiology	63	2.3	2,044	200	32.44
2	Circulation	43	35.5	5,657	198	131.56
3	European Journal of Heart Failure	38	16.9	1,247	148	32.82
4	American Heart Journal	35	3.7	1,322	182	37.77
5	International Journal of Cardiology	30	3.2	693	249	23.10
6	Journal of the American College of Cardiology	30	5.0	158	84	5.27
7	European Heart Journal	23	37.6	1,901	134	82.65
8	ESC Heart Failure	20	3.2	502	104	25.10
9	Journal of the American Heart Association	16	5.0	4,003	86	250.19
10	Journal of Cardiac Failure	14	6.7	480	36	34.29

### 3.4 Keyword analysis

**Figure 6:**
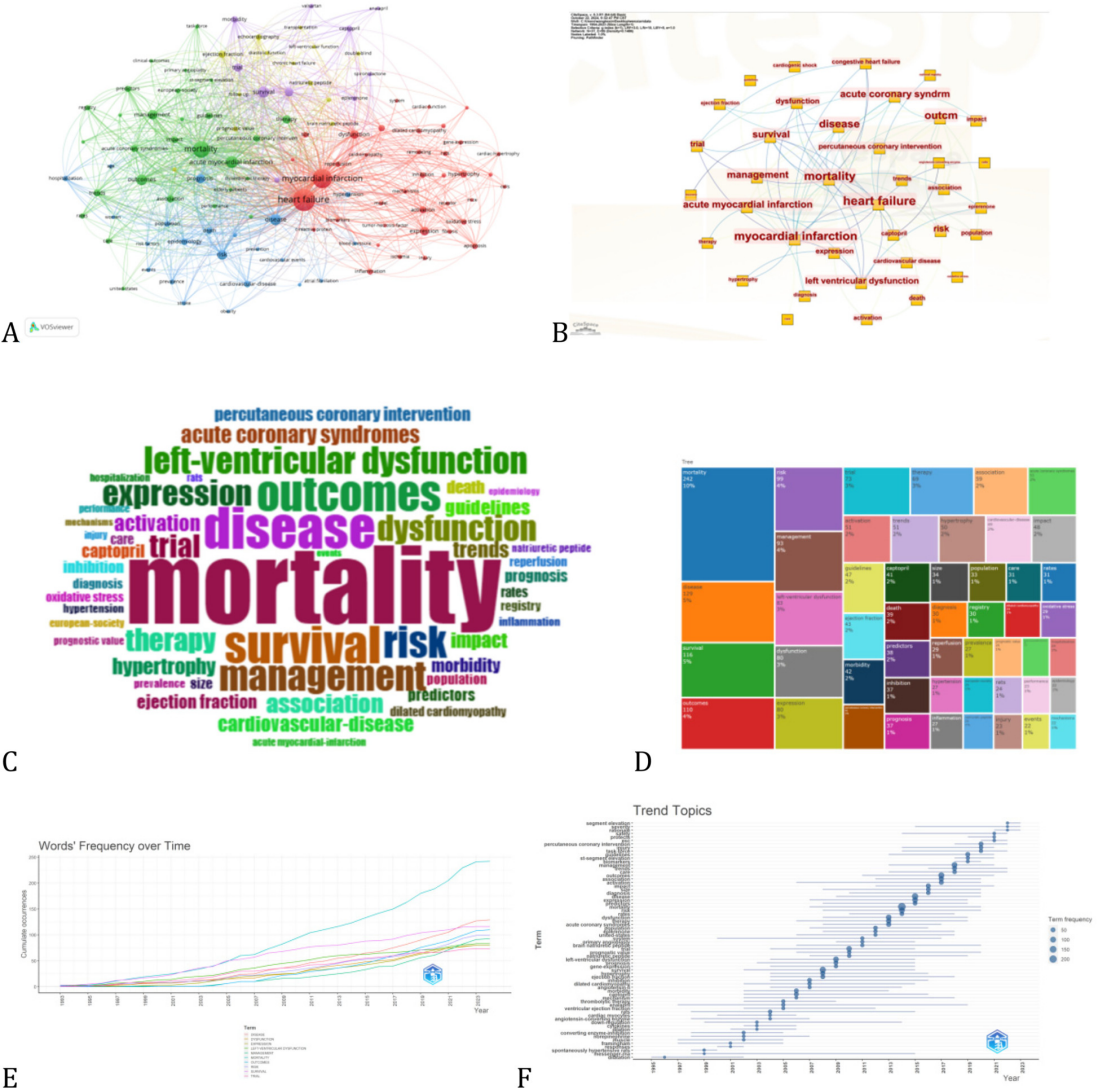
The visualization knowledge maps of keyword, including **(A,B)** keyword network diagram, **(C)** wordcloud, **(D)** treemap, **(E)** word dynamics, and **(F)** trend topics.

Keywords represent the core concepts of an article, allowing us to determine the main research directions and hotspots in the field of myocardial infarction combined with heart failure through co-occurrence analysis. A total of 4,058 unique keywords were extracted from 1,095 articles on this topic. Using VOSviewer, a keyword network was constructed, and keywords occurring more than 15 times were visualized (Figure 6). The top five keywords were heart failure, myocardial infarction, mortality, acute myocardial infarction and disease. Bursty keywords, characterized by their high frequency within a short period, were identified using CiteSpace's timeline graph to track the temporal evolution of research in this field. Table 5 shows that keywords such as mortality, survival, outcomes, and risk have appeared more frequently, particularly the term “mortality,” which has seen a significant increase in recent years. This trend suggests that the severity of myocardial infarction combined with heart failure has escalated and garnered considerable attention.

**Table 5:** The 20 most frequently occurring keywords.

ID	Keyword	Occurrences	Total link strength
1	Heart Failure	516	5,942
2	Myocardial Infarction	384	4,516
3	Mortality	290	3,102
4	Acute Myocardial Infarction	154	1,602
5	Disease	129	1,382
6	Survival	128	1,391
7	Outcomes	118	1,240
8	Risk	101	1,053
9	Management	93	1,014
10	Prognosis	84	939
11	Left-Ventricular Dysfunction	83	959
12	Dysfunction	80	959
13	Expression	80	895
14	Trial	73	738
15	Therapy	72	778
16	Association	59	673
17	Acute Coronary Syndromes	55	656
18	Trends	55	529
19	Activation	51	616
20	Ejection Fraction	51	564

Over the past three decades, research on myocardial infarction (MI) complicated by heart failure (HF) has evolved from acute, pathophysiology-centered investigations to a comprehensive, multidisciplinary approach to chronic cardiac care. In the early 1990s, clinical trials established the mortality benefits of neurohormonal blockade—particularly with ACE inhibitors such as captopril and ramipril (4). By the late 1990s and 2000s, treatment expanded to include β-blockers, ARBs, and aldosterone antagonists, with increasing emphasis on long-term outcomes and comorbidity management, recognizing that survival was only the beginning. In the 2010s, novel therapies such as ARNIs and SGLT2 inhibitors emerged, informed by a deeper understanding of cardiorenal and metabolic interactions beyond the classical RAAS pathway. At the same time, HF care shifted toward a systems-based model—integrating device therapy, revascularization, and the management of common comorbidities like diabetes and kidney disease. Landmark trials like STICH and REVIVED redefined the role of revascularization in ischemic cardiomyopathy, with STICH demonstrating long-term survival benefits from CABG in patients with impaired LV function. These studies reinforced the importance of individualized treatment strategies.

In the 2020s, the field entered a new phase, shaped by precision medicine and artificial intelligence. Modern research integrates genomics, circulating biomarkers, imaging, and machine learning to refine risk stratification and guide therapy. Bibliometric burst analyses show rising interest in terms such as “inflammation,” “biomarker,” “machine learning,” and “microbiome” (5), highlighting key emerging areas. What began as an effort to prevent adverse remodeling has grown into a diverse, interdisciplinary endeavor spanning molecular biology, bioinformatics, and population health. The evolution of research themes from 1993 to 2023—traced through co-occurrence networks and cluster analyses—shows clear transitions: from ACE inhibitors to ARNIs and SGLT2 inhibitors; from short-term survival to long-term, multimorbidity management; from acute coronary care to chronic HF optimization; from ejection fraction metrics to multi-omics biomarkers; and from traditional trials to AI-driven prediction models. Each progression, anchored in both foundational evidence and cutting-edge science, has deepened our understanding and enhanced the care of MI patients with HF. Today, the field offers a more holistic, precise, and hopeful strategy for a once highly fatal condition.

### 3.5 Keywords with citation burst

**Figure 7:**
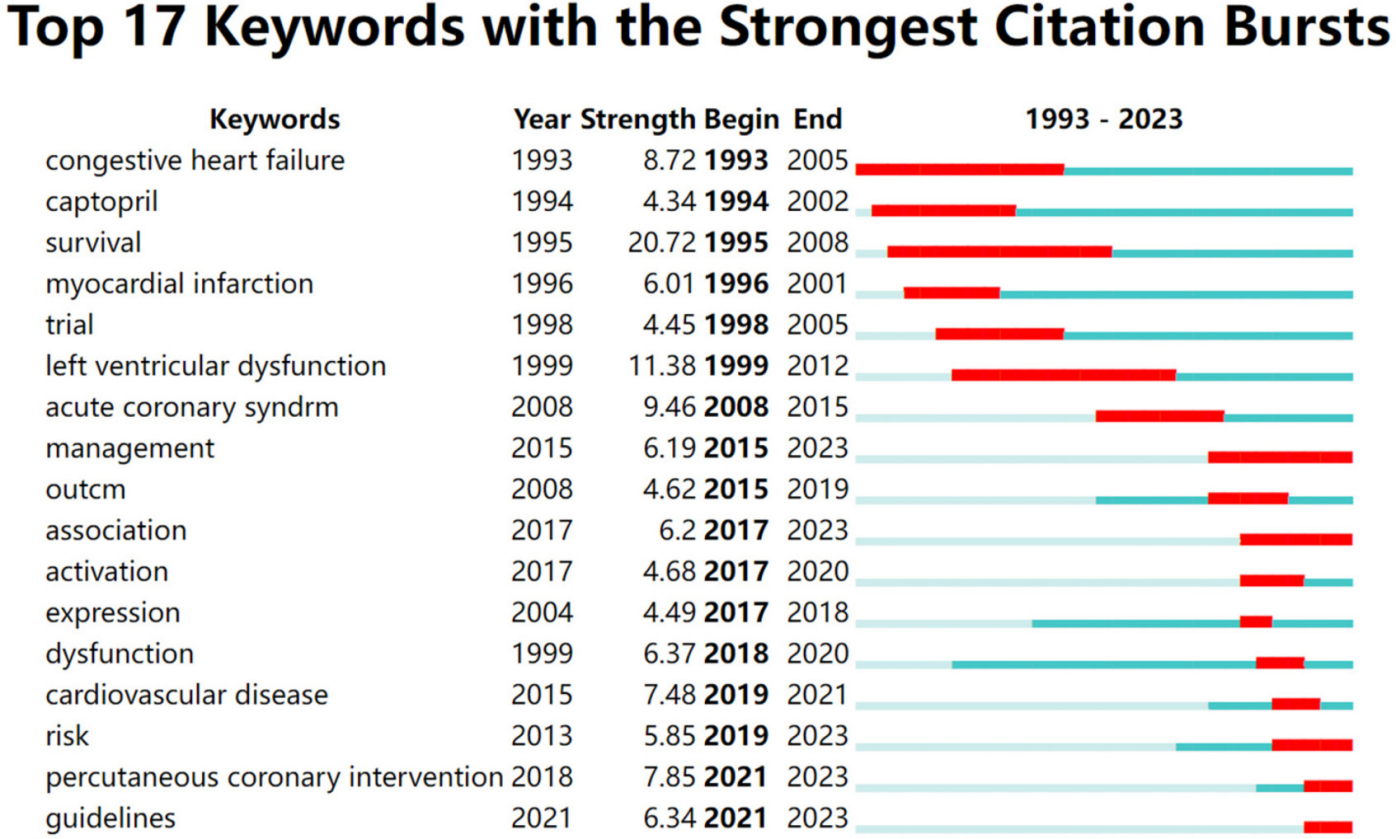
1993–2023 Myocardial infarction combined with heart failure outbreak word.

Figure 7 shows the top 17 keywords with the strongest citation bursts. The keywords of “congestive heart failure” (1993–2005), “captopril” (1994–2002), “survival” (1995–2008), “left ventricular dysfunction” (1999–2012) presented as hotspots for a long period in the past; whereas “management” (2015–2023), “risk” (2019–2023), “guidelines”(2021–2023) and “percutaneous coronary intervention (2021–2023)” have been used more recently, which indicate the future hotspots in the research field of myocardial infarction combined with heart failure.

### 3.6 Analysis of co-cited references

**Figure 8:**
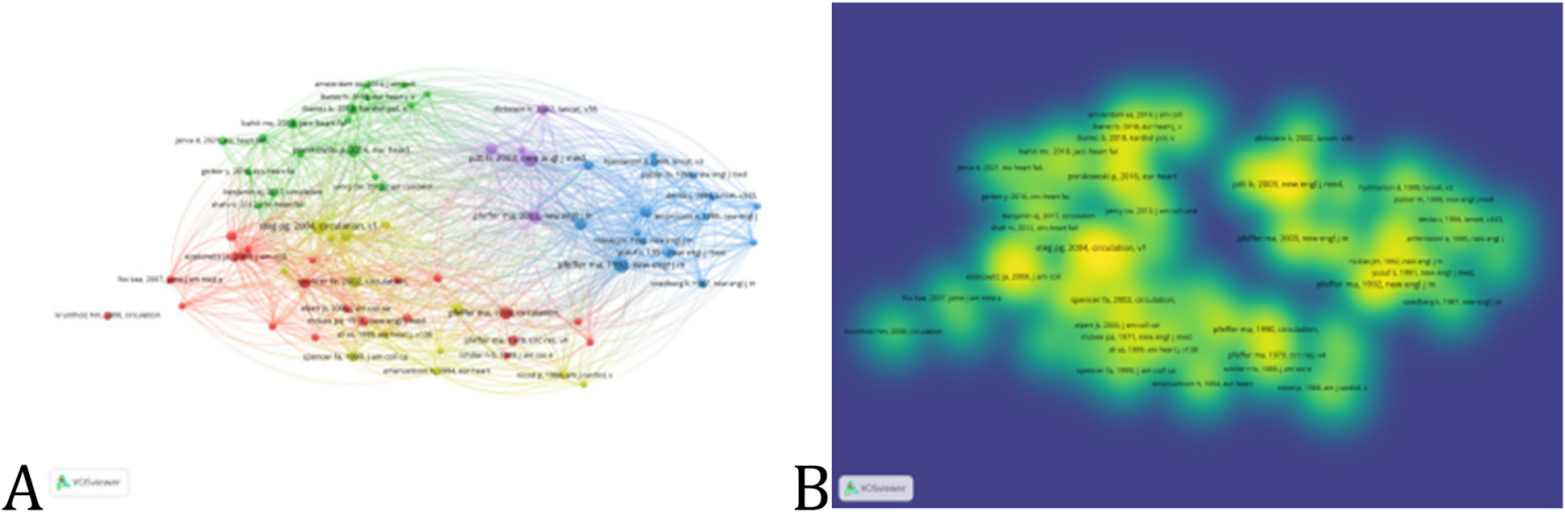
The visualization of co-citation reference, including **(A)** network visualization and **(B)** density visualization.

A co-cited reference arises when two or more articles are cited together by the same article or group of articles. Given that the co-citation relationships among literature evolve over time, analyzing co-cited references can provide insights into the developmental and evolutionary dynamics of a field. Co-citation analysis facilitates the identification of seminal works within the field. We established a threshold of 17 and identified 71 co-cited references (Figure 8). Table 6 highlights the ten most co-cited references. Notably, the articles ranked 8th and 5th have the highest individual citation counts, with 1,895 and 1,770 citations respectively, indicating their significant influence in the field.

**Table 6:** Top ten co-cited references.

ID	Cited reference	Authors	Juornals	Citations	Total link strength
1	Eplerenone, a selective aldosterone blocker, in patients with left ventricular dysfunction after myocardial infarction.	Pitt B, 2003	New Engl J Med	76	138
2	Impact of time to treatment on mortality after prehospital fibrinolysis or primary angioplasty—Response	Steg PG, 2004	Circulation	70	107
3	Effect of captopril on mortality and morbidity in patients with left ventricular dysfunction after myocardial infarction. Results of the survival and ventricular enlargement trial. The SAVE Investigators.	Pfeffer MA, 1992	New Engl J Med	68	137
4	2016 ESC Guidelines for the diagnosis and treatment of acute and chronic heart failure: the Task Force for the diagnosis and treatment of acute and chronic heart failure of the European Society of Cardiology (ESC) Developed with the special contribution of the Heart Failure Association (HFA) of the ESC.	Ponikowski P, 2016	Eur Heart J	57	37
5	Valsartan, captopril, or both in myocardial infarction complicated by heart failure, left ventricular dysfunction, or both.	Pfeffer MA, 2003	New Engl J Med	52	115
6	Ventricular remodeling after myocardial infarction. Experimental observations and clinical implications.	Pfeffer MA, 1990	Circulation	48	29
7	Effect of carvedilol on outcome after myocardial infarction in patients with left-ventricular dysfunction: the CAPRICORN randomised trial	Dargie HJ, 2001	Lancet	45	114
8	Effect of Ramipril on mortality and morbidity of survivors of acute myocardial-infarction with clinical-evidence of heart-failure	Ball SG, 1993	Lancet	43	107
9	A clinical trial of the angiotensin-converting-enzyme inhibitor trandolapril in patients with left ventricular dysfunction after myocardial infarction. Trandolapril Cardiac Evaluation (TRACE) Study Group.	Kober L, 1995	New Engl J Med	43	96
10	Hospital outcomes in patients presenting with congestive heart failure complicating acute myocardial infarction—A report from the Second National Registry of Myocardial Infarction (NRMI-2)	Wu AH, 2002	J Am Coll Cardiol	43	88

## 4 Discussion

This study conducted a bibliometric analysis of myocardial infarction combined with heart failure spanning the years 1993–2023. We utilized software such as CiteSpace, VOSviewer, and RStudio to quantitatively visualize the evolution and research hotspots in this field. The results reveal a notable rise in the number of publications over the past three decades. We then carried out a comprehensive quantitative analysis of various factors, such as countries, authors, institutions, journals, keywords, and references. The United States stood out as the most productive and influential country in this field, maintaining strong collaborations with other nations. U.S. institutions ranked high in both the volume and quality of publications. Notably, authors with the highest publication counts are predominantly from the United States, France, Denmark, and other countries. Among them, Prof. Faiez Zannad from the University of Lorraine, France, has the highest publication count, the strongest collaboration links, and is considered one of the most influential authors. In terms of journals, CIRCULATION stands out as one of the most influential in this field, boasting a high volume of publications, impact factor, average citation frequency, and H-index. Furthermore, the analysis of emerging terms indicates that future research is likely to focus on the screening and treatment of myocardial infarction combined with heart failure. Finally, through co-citation analysis, we identified key classic literature in this field.

Post-myocardial infarction (MI) heart failure (HF) remains a major clinical challenge, driven by complex molecular and cellular mechanisms. Central to its development is adverse left ventricular remodeling, which begins with widespread cardiomyocyte necrosis/apoptosis and compensatory hypertrophy, followed by excessive extracellular matrix deposition (fibrosis), ultimately impairing cardiac function. The initial ischemic insult activates a sterile inflammatory response via damage-associated molecular patterns (DAMPs), engaging innate immune pathways such as Toll-like receptors and inflammasomes. These trigger nuclear factor-κB (NF-κB) activation and the release of inflammatory cytokines like IL-1β, IL-6, and TNF-α (6). Although essential for clearing necrotic tissue, sustained inflammation aggravates myocardial damage and remodeling. Proteomic analysis in post-MI HF patients has highlighted differentially expressed proteins linked to the NF-κB pathway, emphasizing its central role in disease progression (7). NF-κB–driven gene expression promotes cytokines, chemokines, and matrix metalloproteinases that exacerbate tissue injury, while experimental NF-κB inhibition alleviates remodeling in animal models.

In parallel, neurohormonal activation—particularly via the renin–angiotensin–aldosterone system (RAAS)—contributes significantly to HF. Angiotensin II drives vasoconstriction, hypertrophy, and fibroblast activation, whereas ACE2 exerts a counter-regulatory effect by degrading Ang II into the protective Ang-(1–7) peptide. Under ischemic conditions, Chen et al. (8) found that p38 MAPK-mediated activation of ADAM17 leads to ACE2 shedding, removing its cardioprotective role and intensifying RAAS-mediated fibrosis and hypertrophy. ADAM17 upregulation correlates with increased infarct size and worse remodeling, while its inhibition improves cardiac outcomes in mice. Moreover, ADAM17 enhances inflammation by cleaving cytokine precursors and receptors (e.g., pro-TNF-α, IL-6R), reinforcing its role as a molecular driver of post-MI HF.

During the healing phase, TGF-β–induced activation of cardiac fibroblasts results in scar formation. While necessary for structural integrity, excessive or diffuse fibrosis increases ventricular stiffness and promotes HF, even with preserved ejection fraction. Recent transcriptomic studies have identified four key genes (KLRC2, SNORD105, SNORD45B, RNU5A-1) linked to post-MI HF, suggesting immune dysregulation involvement. Non-coding RNAs, particularly microRNAs, also regulate remodeling: let-7c promotes fibrosis and apoptosis, whereas let-7i suppresses inflammation and collagen production. Dysregulation of these miRNAs can drive maladaptive remodeling, and their therapeutic modulation has shown promise in reducing fibrosis and improving function in preclinical models.

Predictors, predictive models, and biomarkers of heart failure (HF) in combination with myocardial infarction have garnered significant research attention in recent years. A study identified two potential immune-related hub genes (IRHGs), CXCR5 and FOS, through a bioinformatics approach. Functional enrichment analysis showed that these IRHGs were closely associated with immune system processes, particularly the interleukin-17 and nuclear factor-κB signaling pathways, which are critical in HF pathogenesis. The study demonstrated that these two IRHGs were differentially expressed in the HF group and could serve as predictors of heart failure after long-term myocardial infarction (9). Another study recognized serum high-mobility group box 1 (HMGB1) as a biomarker for acute myocardial infarction with heart failure (10). Additionally, RT-qPCR analysis identified FOS, DUSP1, CXCL8, and NFKBIA as potential biomarkers for identifying AMI patients at risk of developing HF (11). Moreover, another study revealed that combining glycan antigen 125 with N-terminal B-type natriuretic peptide precursor enhanced the prediction of acute heart failure following ST-segment elevation myocardial infarction (12).

The treatment of myocardial infarction combined with heart failure remains a relatively underexplored area. Western medicine primarily relies on traditional anti-coronary drugs aimed at improving ventricular remodeling, such as sacubitril/valsartan and ACEI/ARB. Recently, research into traditional Chinese medicine has gained attention. For instance, the Xin-Li formula has been shown to alleviate heart failure caused by hyperlipidemia and myocardial infarction in rats through Treg immunomodulation and inhibition of the NLRP3 inflammasome (13). Similarly, Linggui Zhugan decoction delays ventricular remodeling in rats with chronic heart failure after myocardial infarction via the Wnt/β-catenin signaling pathway (14), and Nuanxinkang prevents chronic heart failure induced by myocardial infarction by enhancing PINK1/Parkin-mediated mitophagy (15). However, most of these studies are still limited to animal models. Research on therapeutic protein targets has also progressed alongside advances in cardiovascular disease understanding. For example, C1q/tumor necrosis factor-related protein 12 (CTRP12) has been found to reduce heart failure after myocardial infarction by modulating the TAK1-p38 MAPK/JNK signaling pathway, suggesting its potential as a therapeutic target for post-MI heart failure (16). Additionally, the apelin receptor (APJ), a widely expressed G protein-coupled receptor, holds promise as a therapeutic target for peptide analogs in treating myocardial infarction and hypertension-induced heart failure (17).

Studies have identified several drugs and their components that exhibit therapeutic effects on myocardial infarction combined with heart failure. For example, levosimendan has demonstrated effectiveness in enhancing cardiac function, improving hemodynamics, and reducing systemic inflammation in patients with acute myocardial infarction and heart failure (18). Resveratrol, a diphenylethylene polyphenol, exhibits beneficial biological properties against chronic diseases. Recent evidence suggests that resveratrol provides cardioprotective effects in animal models of atherosclerosis, ischemic heart disease, and heart failure (19).

## 5 Limitation

This study has several limitations: First, although WoSCC is the most widely used search database, some relevant articles might still have been missed. Second, the continuous updating of the database introduces a time lag in the data, including metrics like article counts, citation numbers, and H-index values. Third, variations in the quality of the collected articles may affect the reliability of the findings.

## 6 Conclusion

This study conducted a systematic review of 1,095 papers published from January 1993 to December 2023, highlighting research progress, key topics, and trends in myocardial infarction combined with heart failure. Over the last three decades, substantial progress has been achieved in understanding the factors influencing this condition, its epidemiology, and its adverse clinical outcomes. Future studies should prioritize exploring the mechanisms of comorbidities and developing effective interventions.

## Data Availability

The original contributions presented in the study are included in the article/Supplementary Material, further inquiries can be directed to the corresponding author.
