# Supplementary figures and images for: Bibliometric study and visualization of myocardial infarction combined with heart failure, 1993–2023
Source: Front Cardiovasc Med. 2025 Aug 8;12:1555748. doi: 10.3389/fcvm.2025.1555748 (PMC12370733; doi:10.3389/fcvm.2025.1555748)

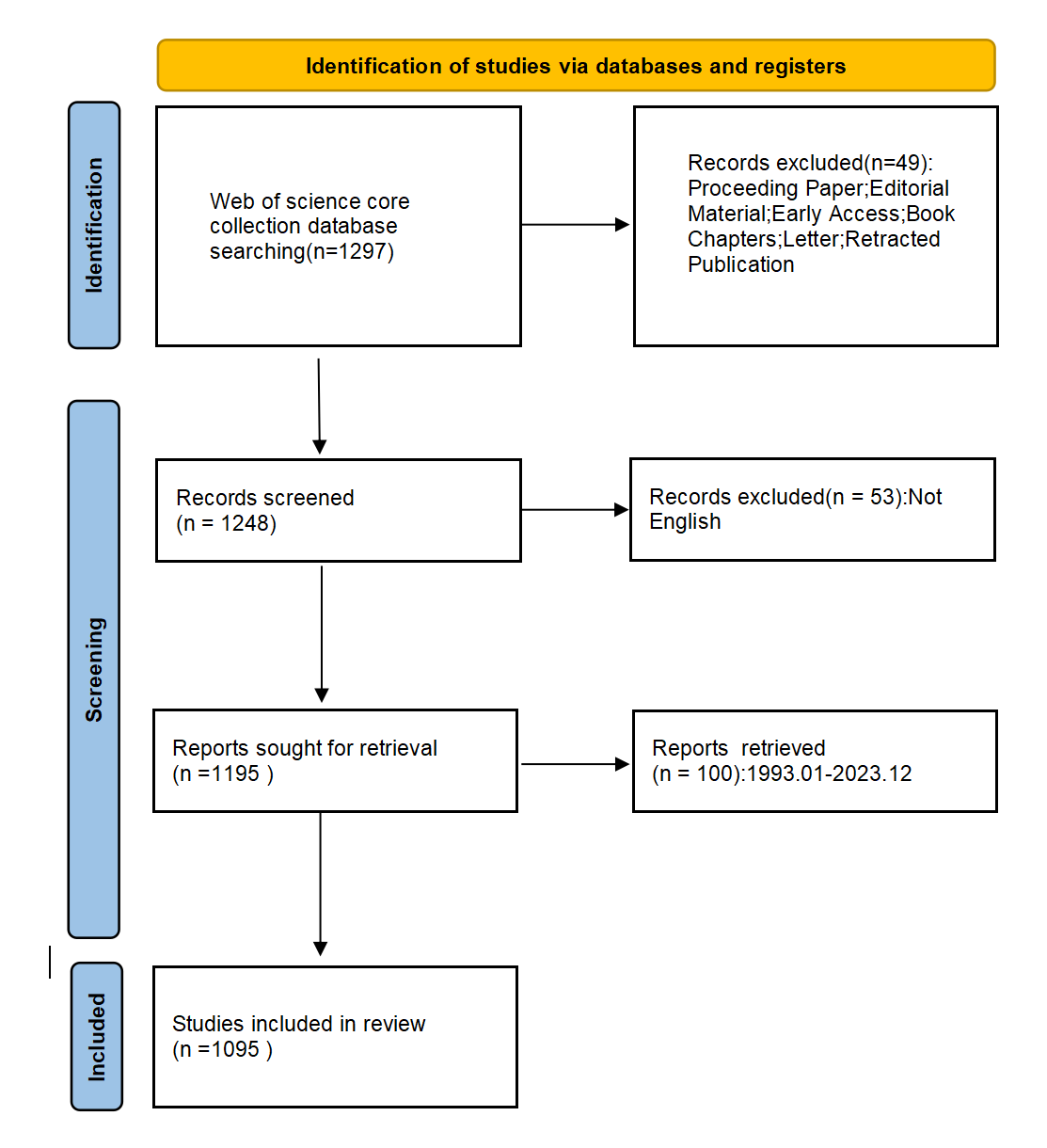

Supplement: Supplementary file 1 [file Datasheet1.zip › figure/1.png]

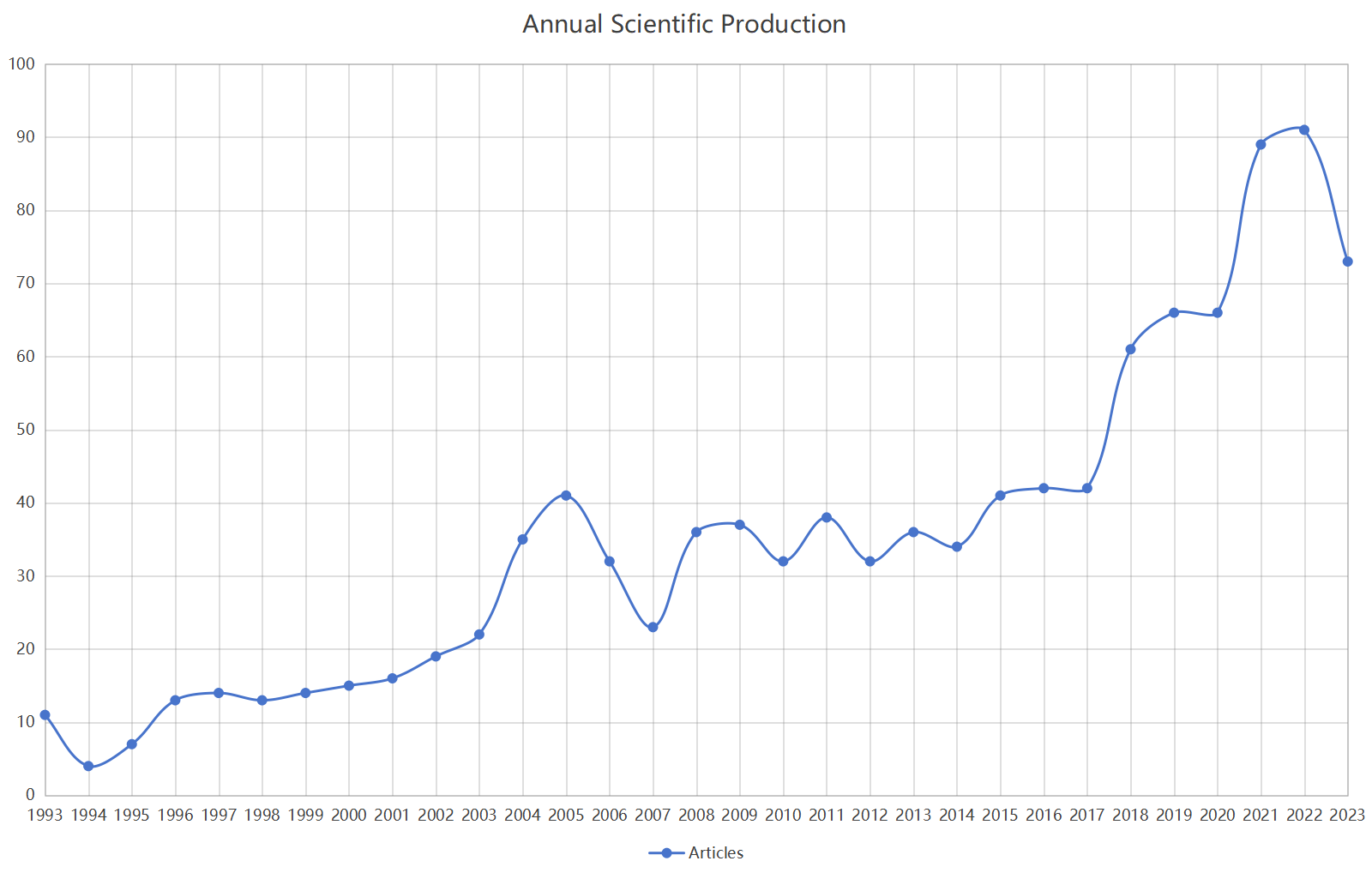

Supplement: Supplementary file 1 [file Datasheet1.zip › figure/2.png]

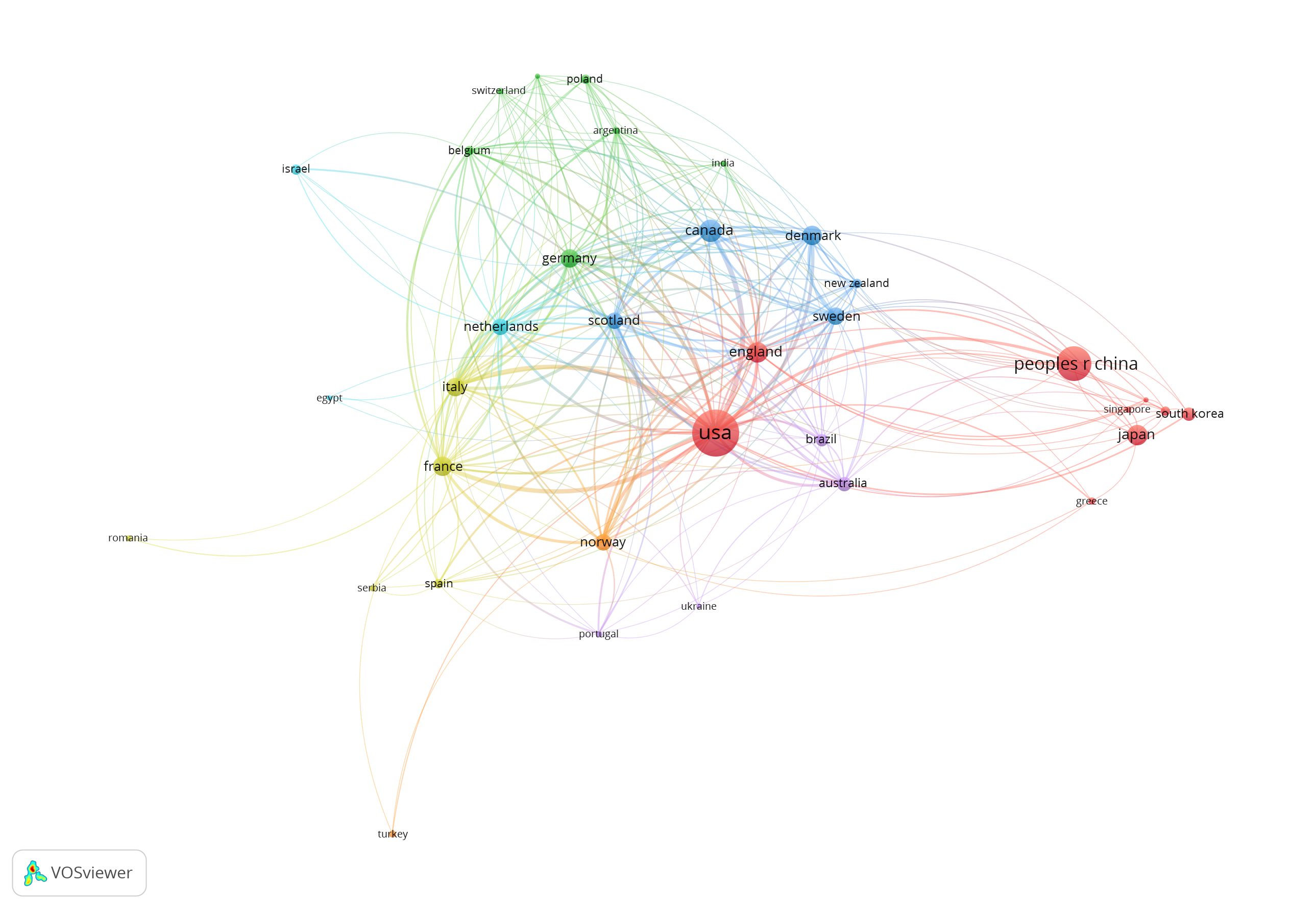

Supplement: Supplementary file 1 [file Datasheet1.zip › figure/3-A.tiff]

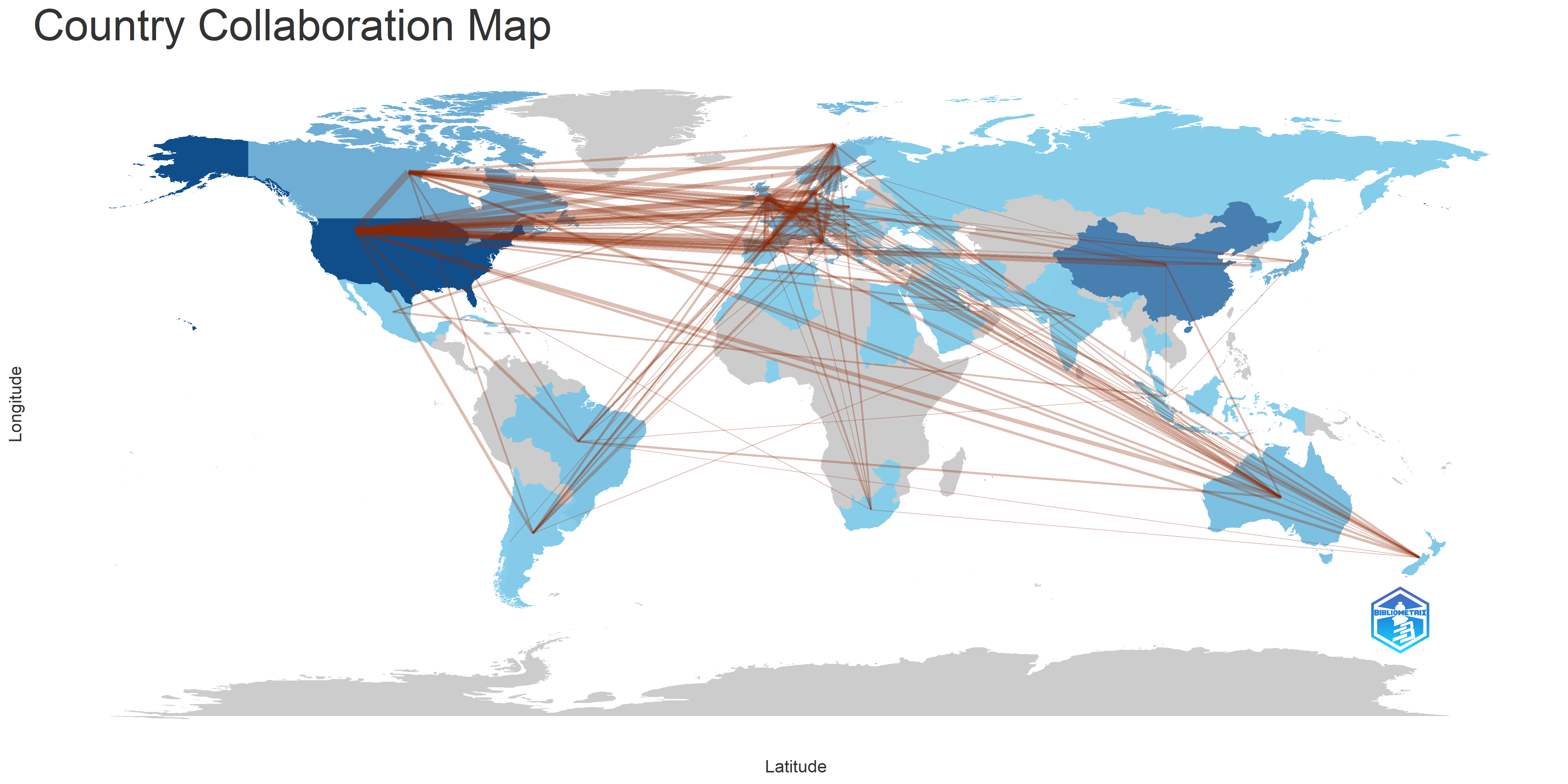

Supplement: Supplementary file 1 [file Datasheet1.zip › figure/3-B.png]

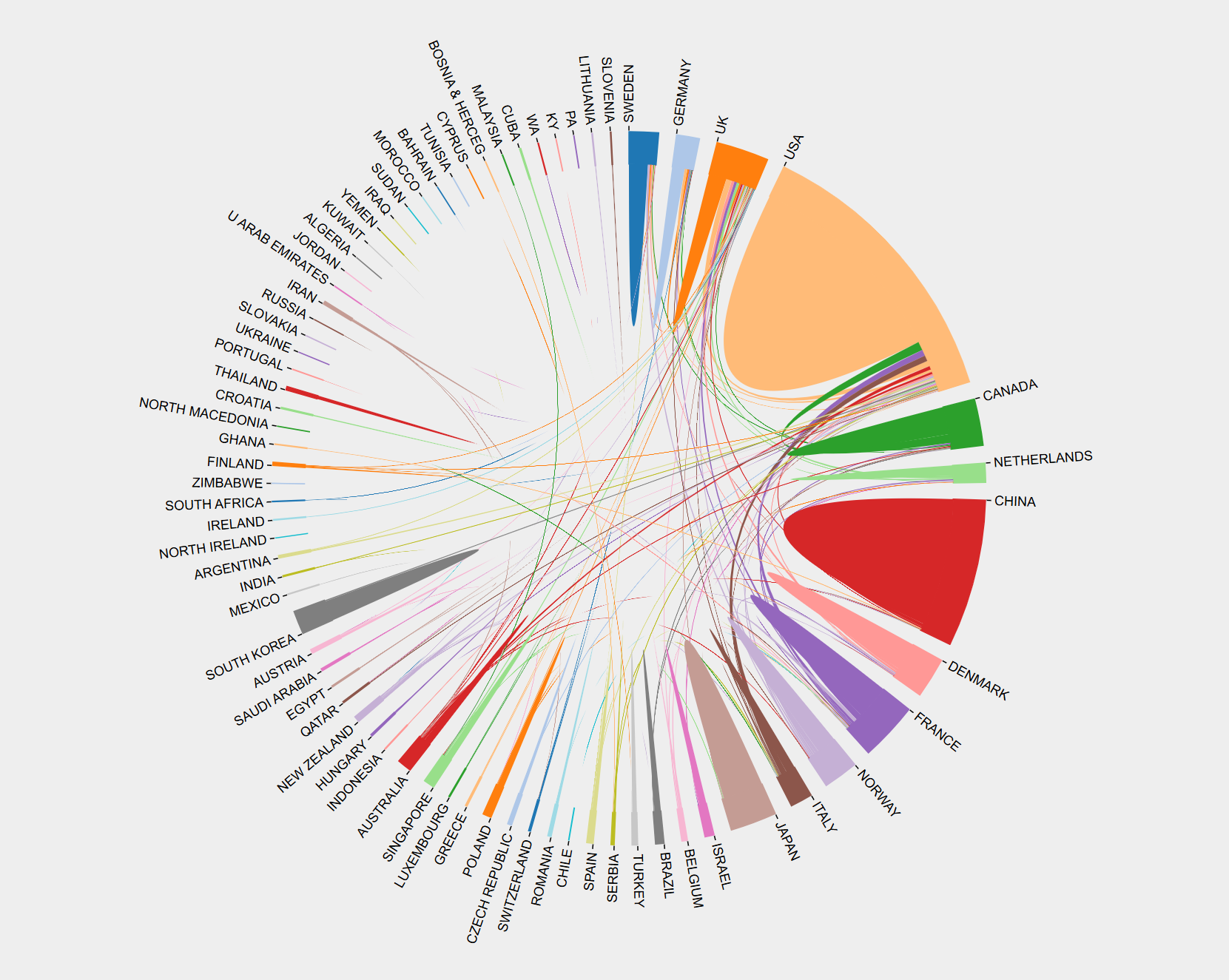

Supplement: Supplementary file 1 [file Datasheet1.zip › figure/3-C.png]

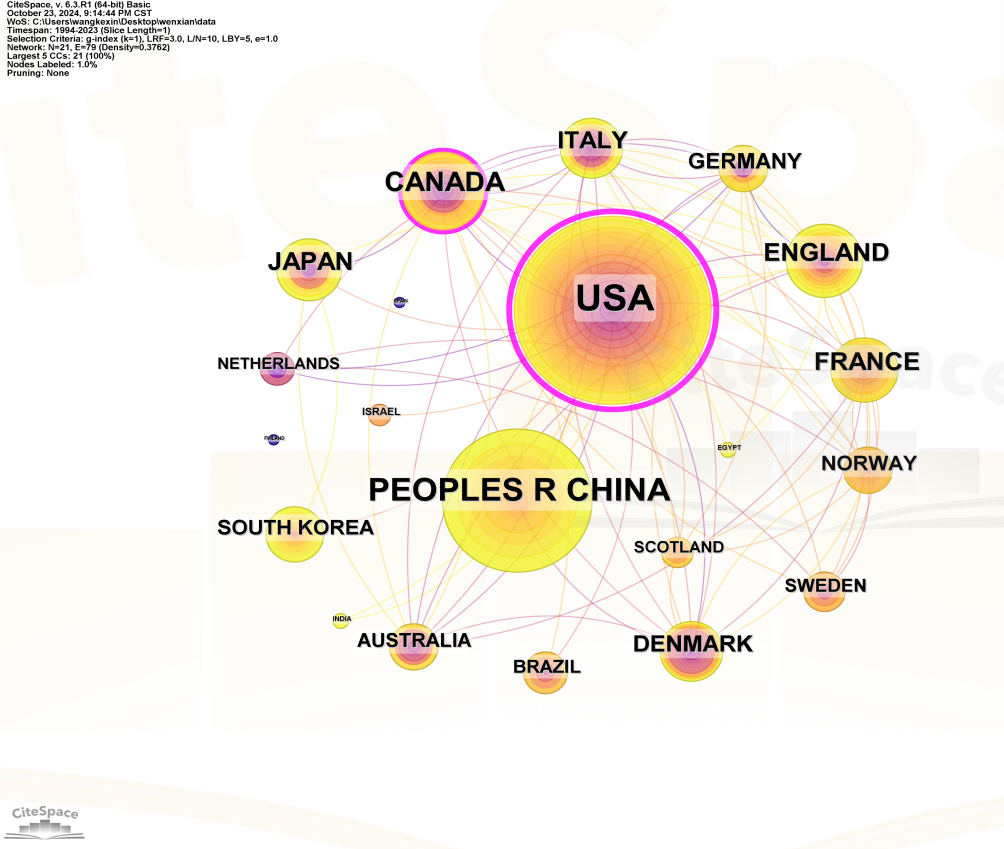

Supplement: Supplementary file 1 [file Datasheet1.zip › figure/3-D.png]

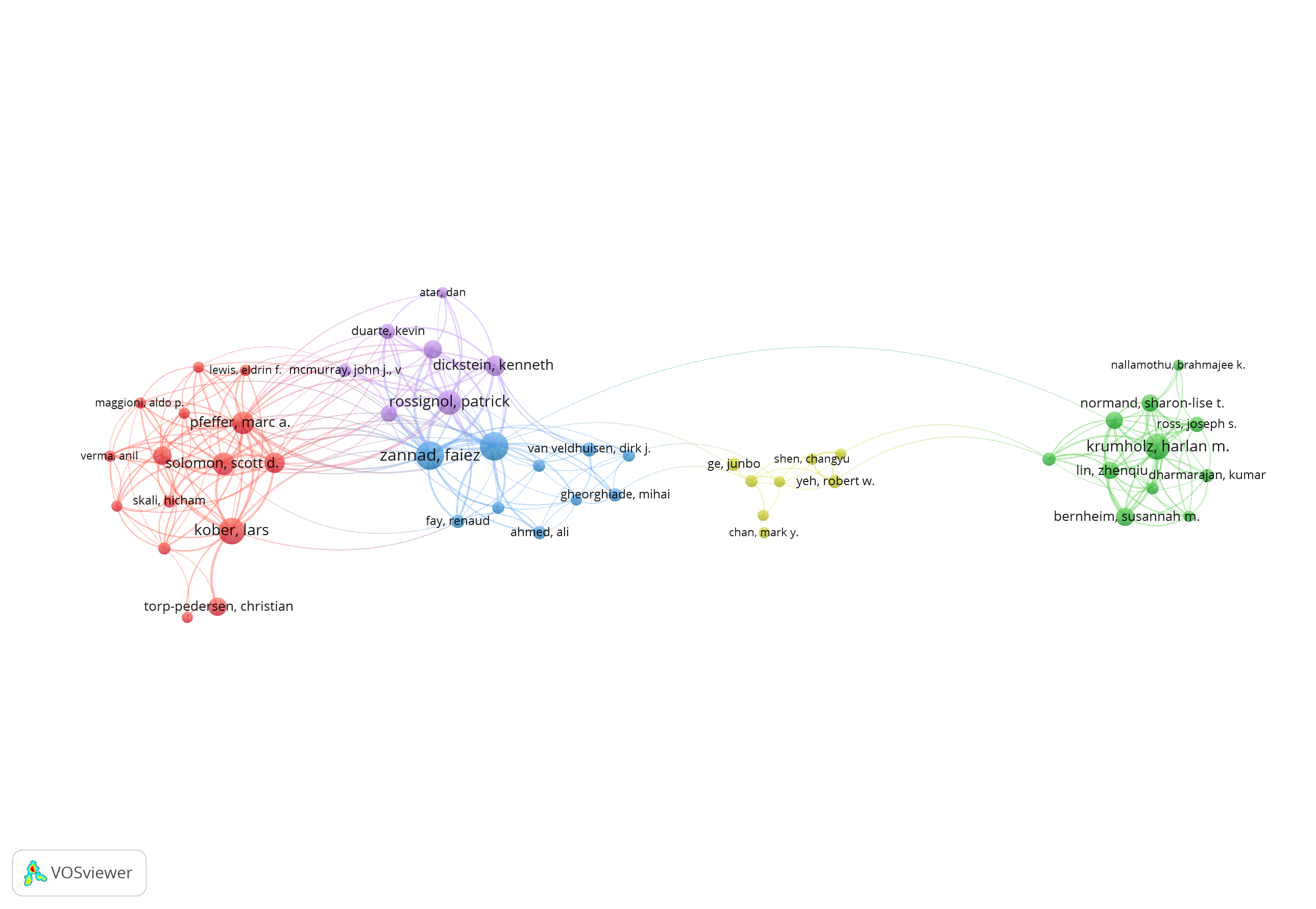

Supplement: Supplementary file 1 [file Datasheet1.zip › figure/4-A.tiff]

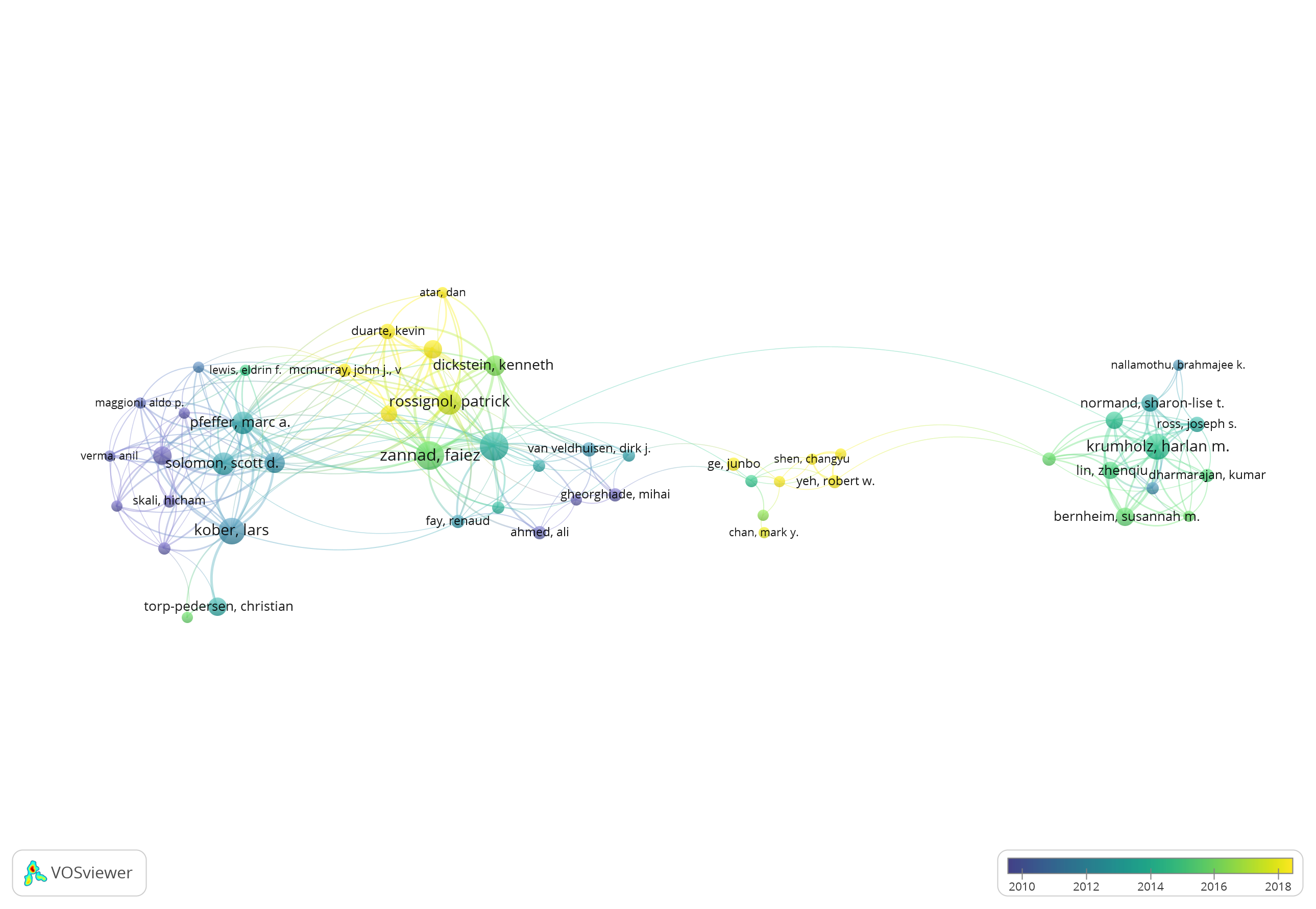

Supplement: Supplementary file 1 [file Datasheet1.zip › figure/4-B.tiff]

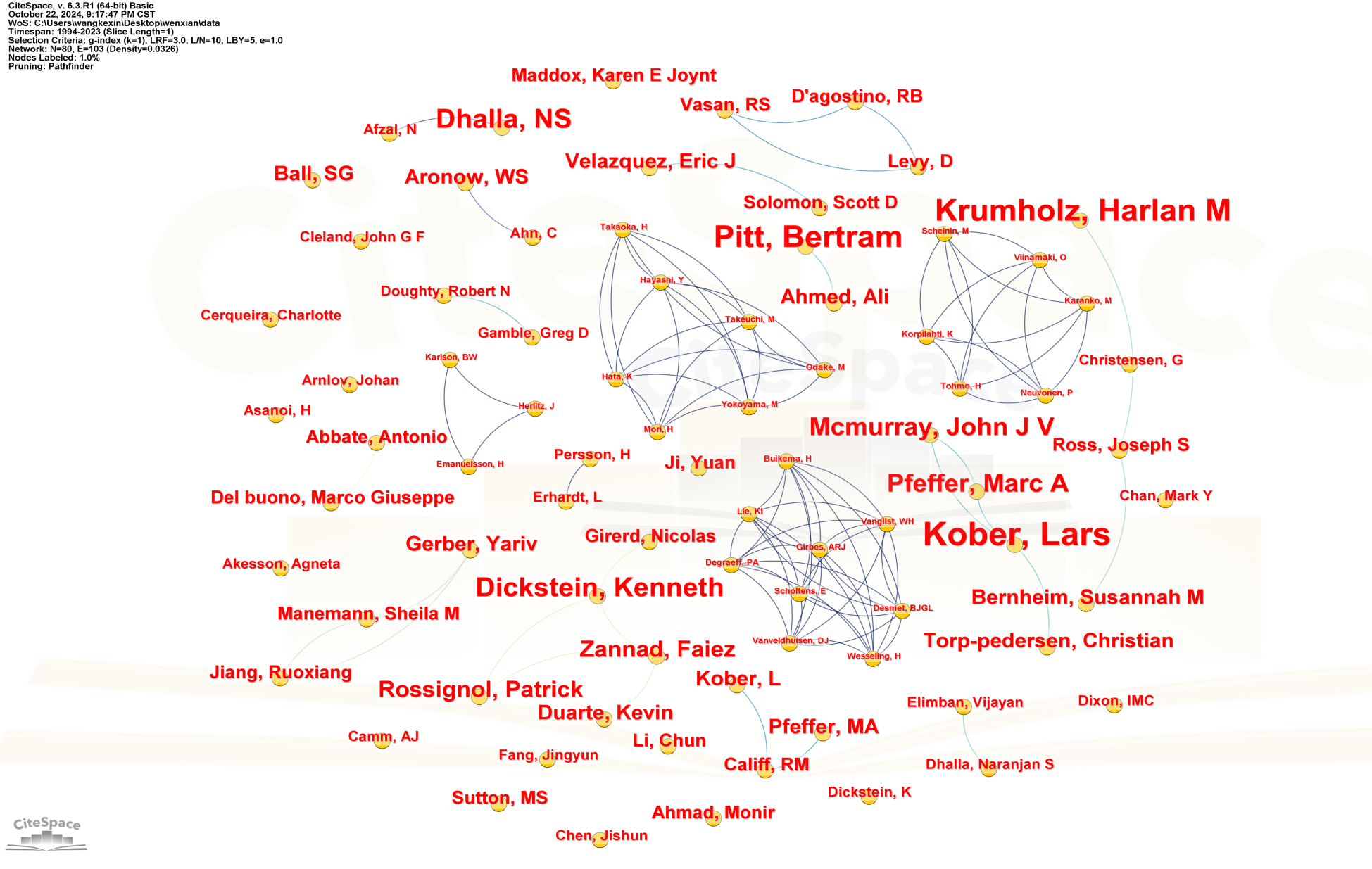

Supplement: Supplementary file 1 [file Datasheet1.zip › figure/4-C.png]

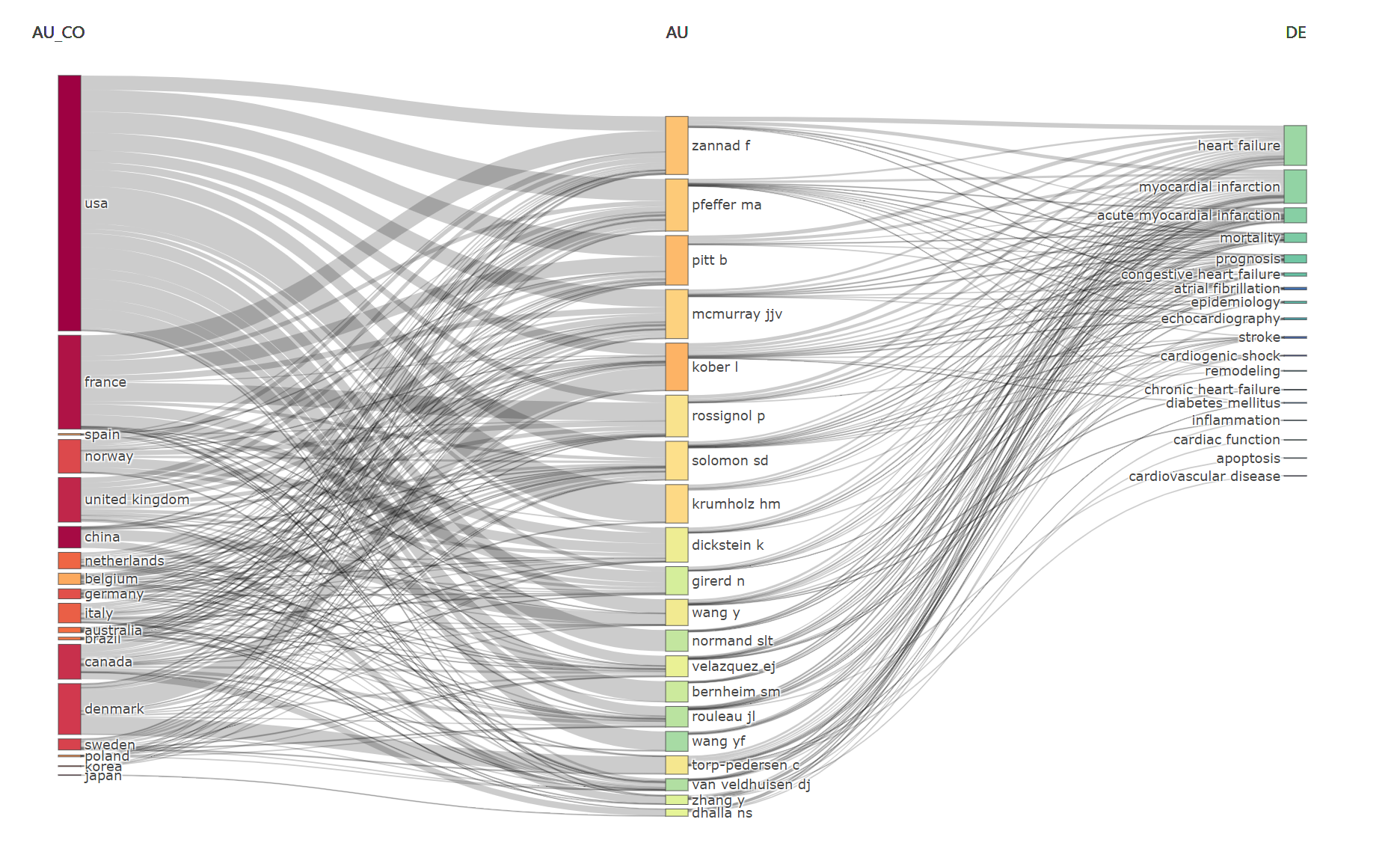

Supplement: Supplementary file 1 [file Datasheet1.zip › figure/4-D.png]

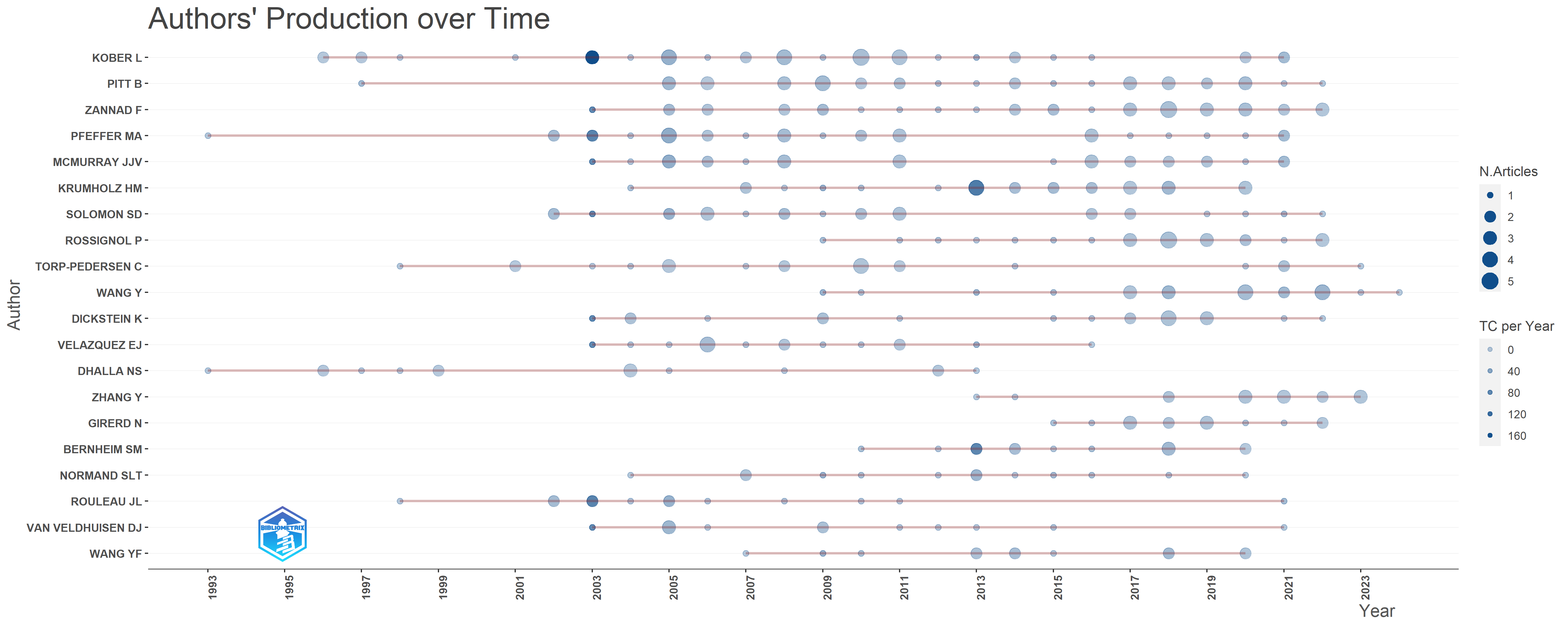

Supplement: Supplementary file 1 [file Datasheet1.zip › figure/4-E.png]

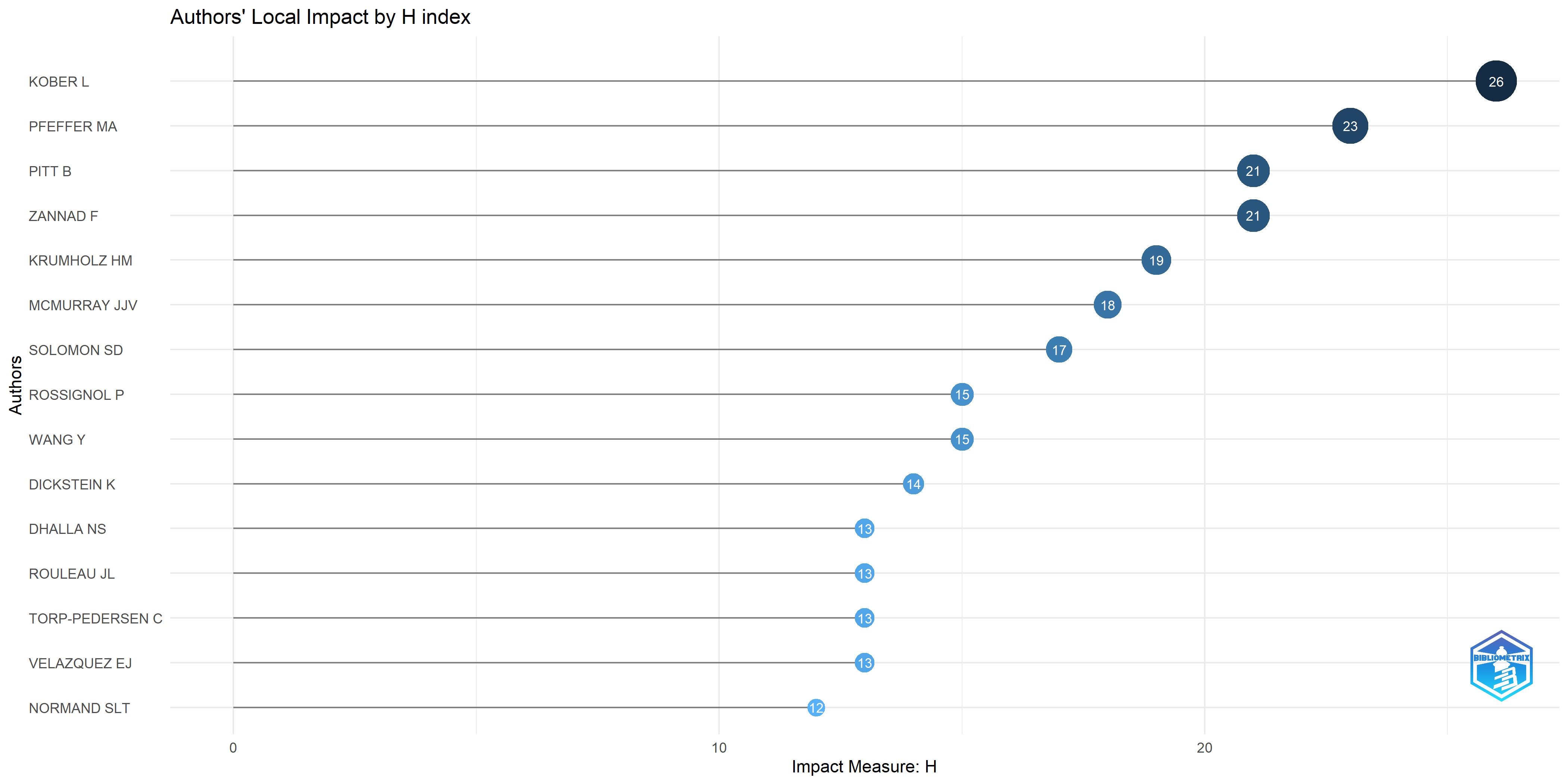

Supplement: Supplementary file 1 [file Datasheet1.zip › figure/4-F.png]

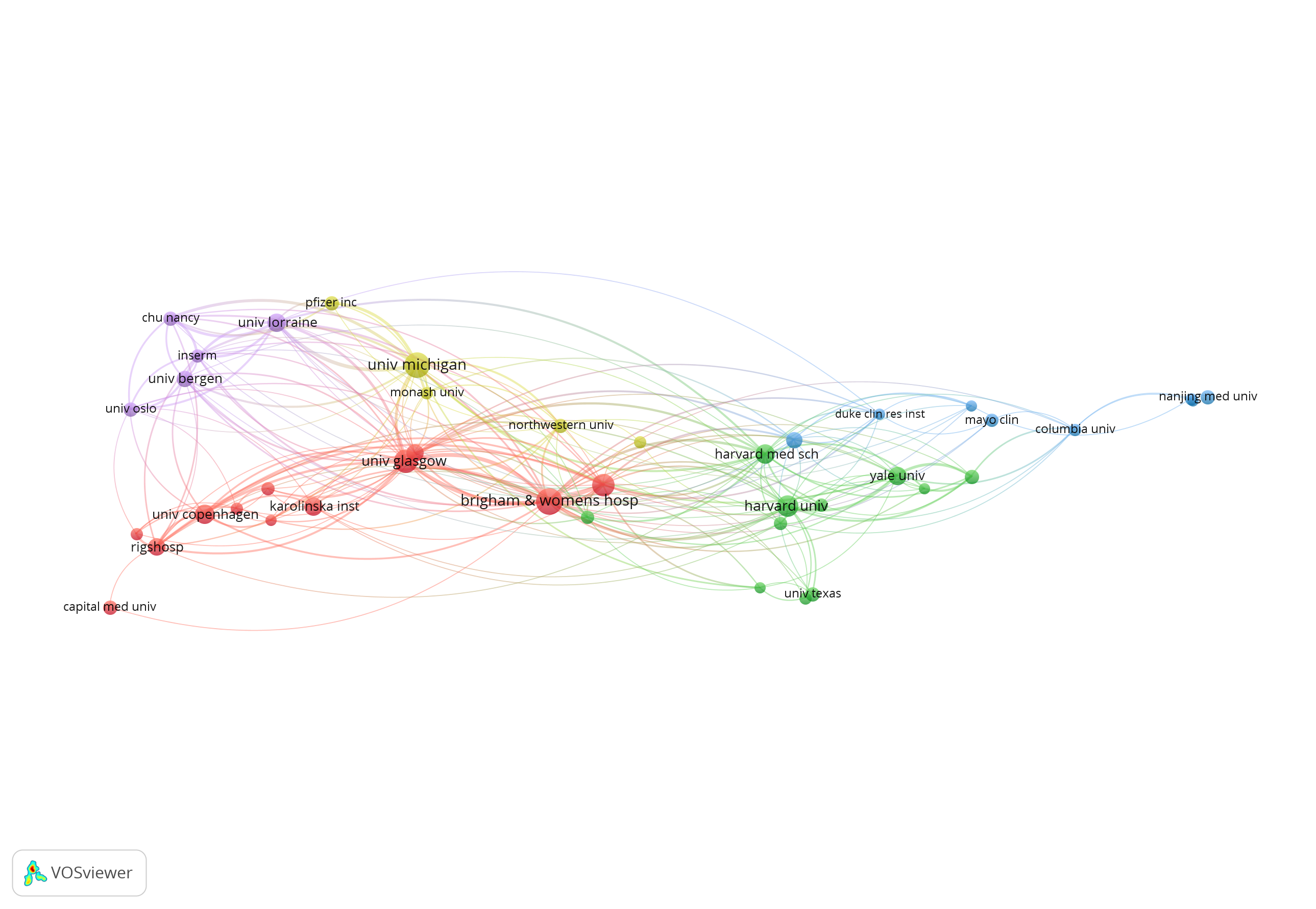

Supplement: Supplementary file 1 [file Datasheet1.zip › figure/5-A.tiff]

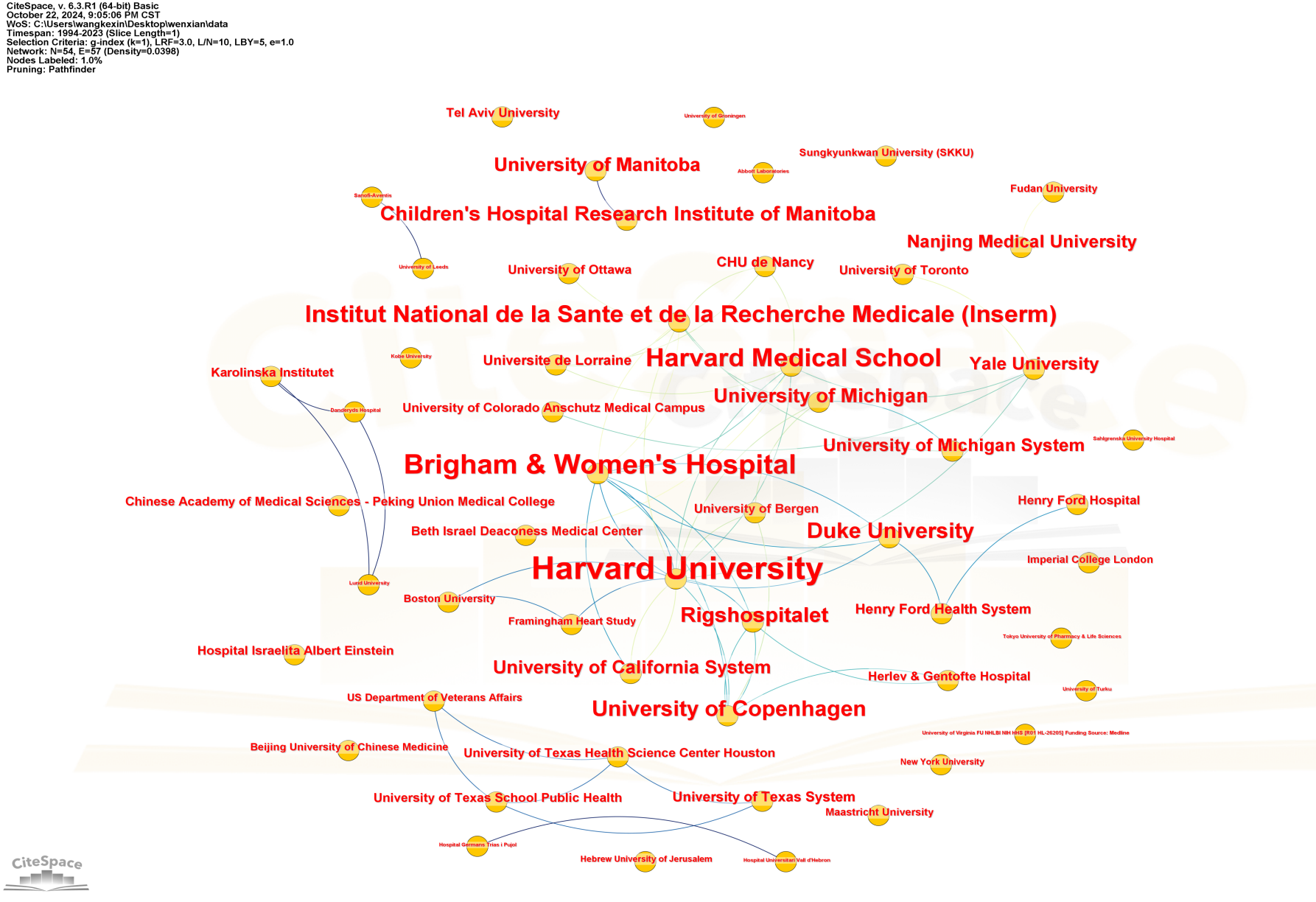

Supplement: Supplementary file 1 [file Datasheet1.zip › figure/5-B.png]

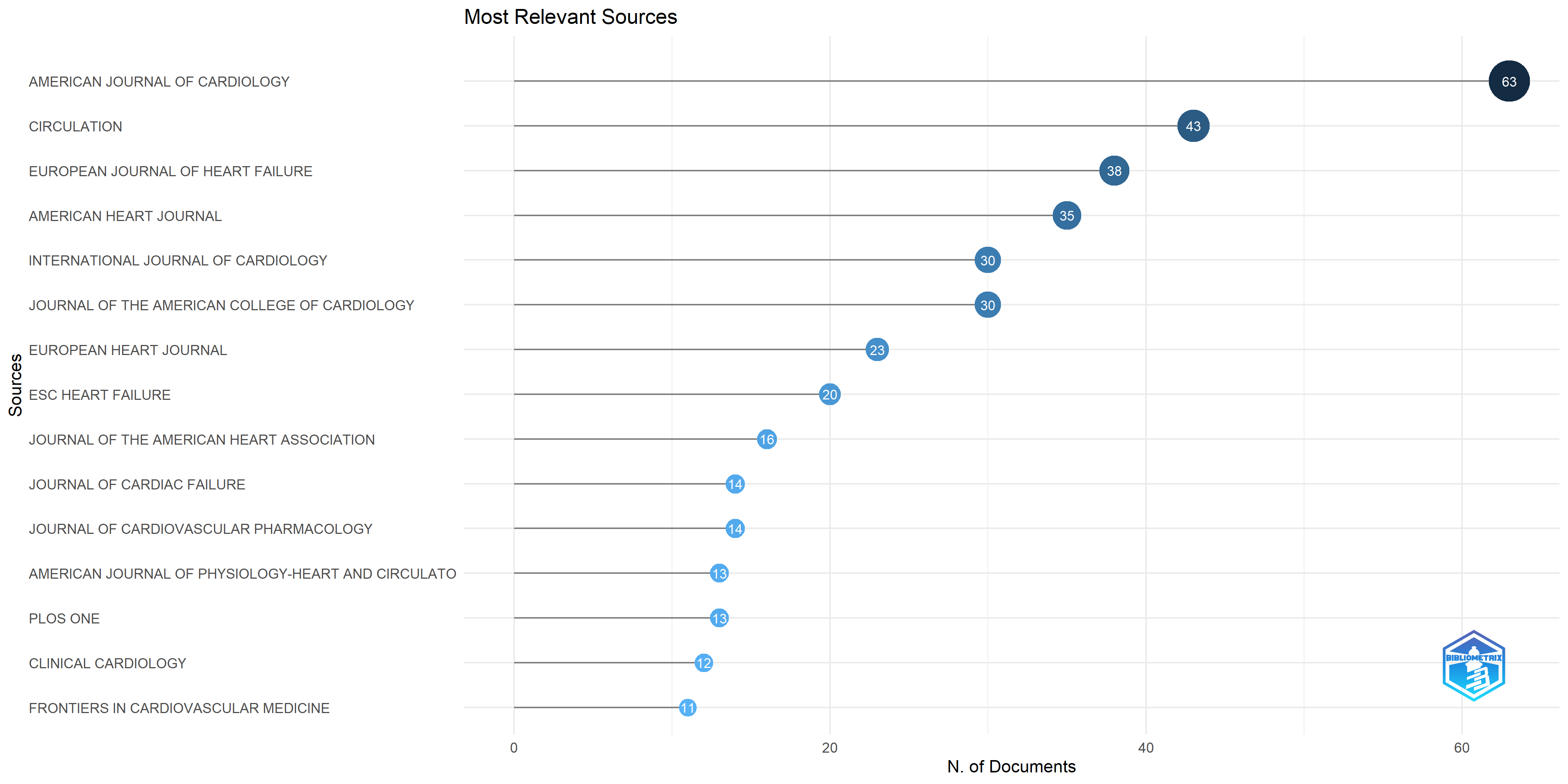

Supplement: Supplementary file 1 [file Datasheet1.zip › figure/6-A.png]

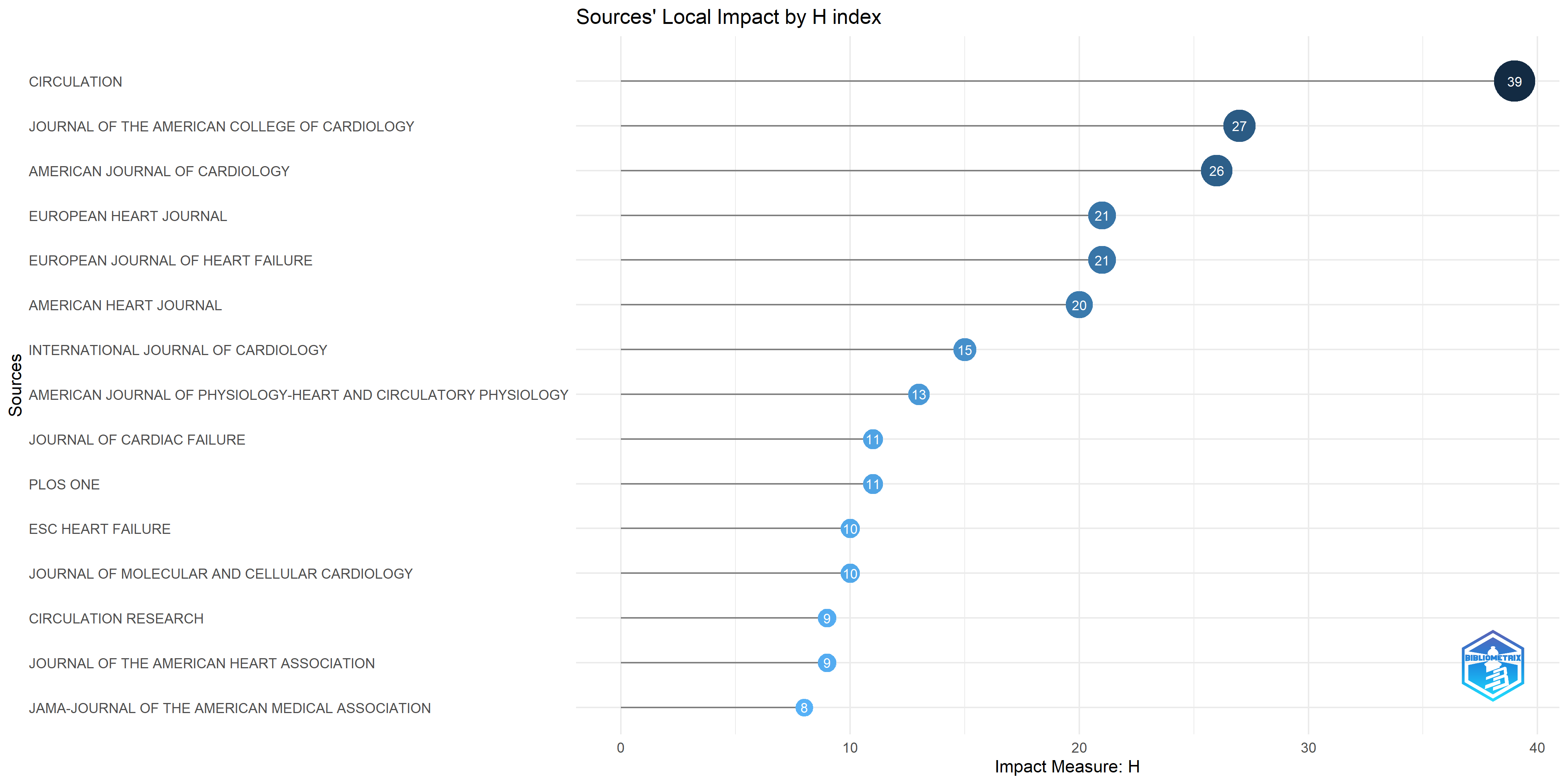

Supplement: Supplementary file 1 [file Datasheet1.zip › figure/6-B.png]

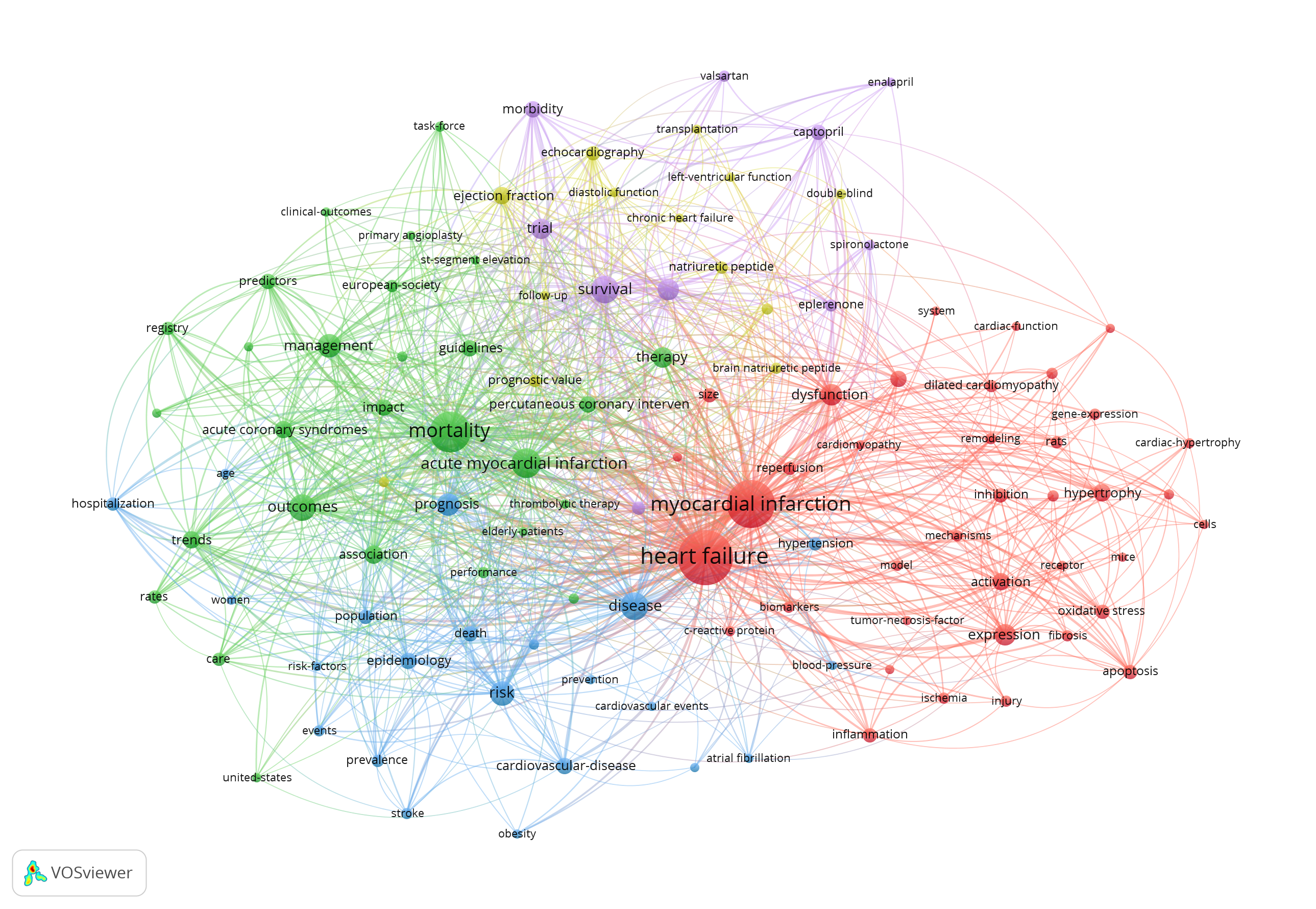

Supplement: Supplementary file 1 [file Datasheet1.zip › figure/7-A.tiff]

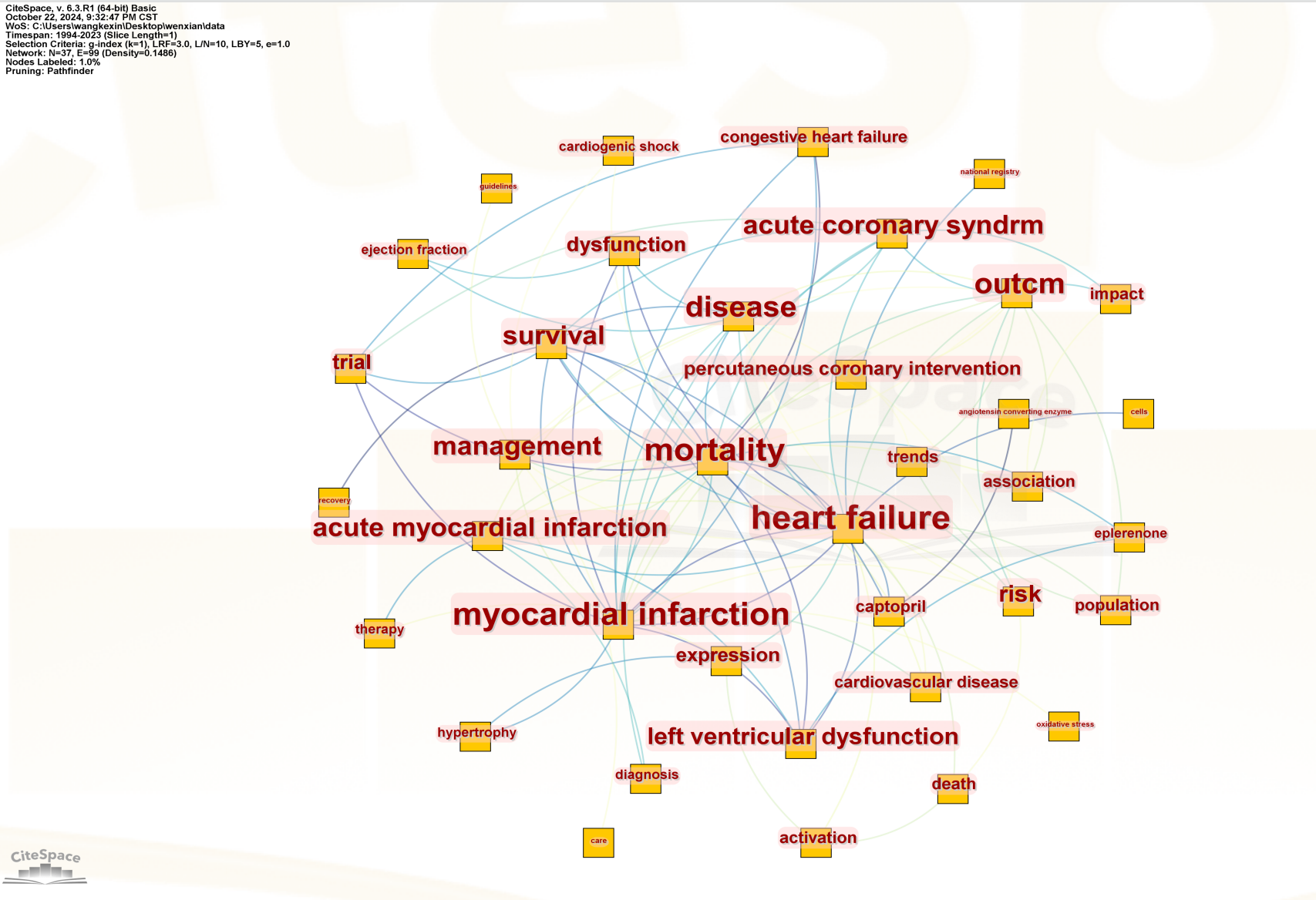

Supplement: Supplementary file 1 [file Datasheet1.zip › figure/7-B.png]

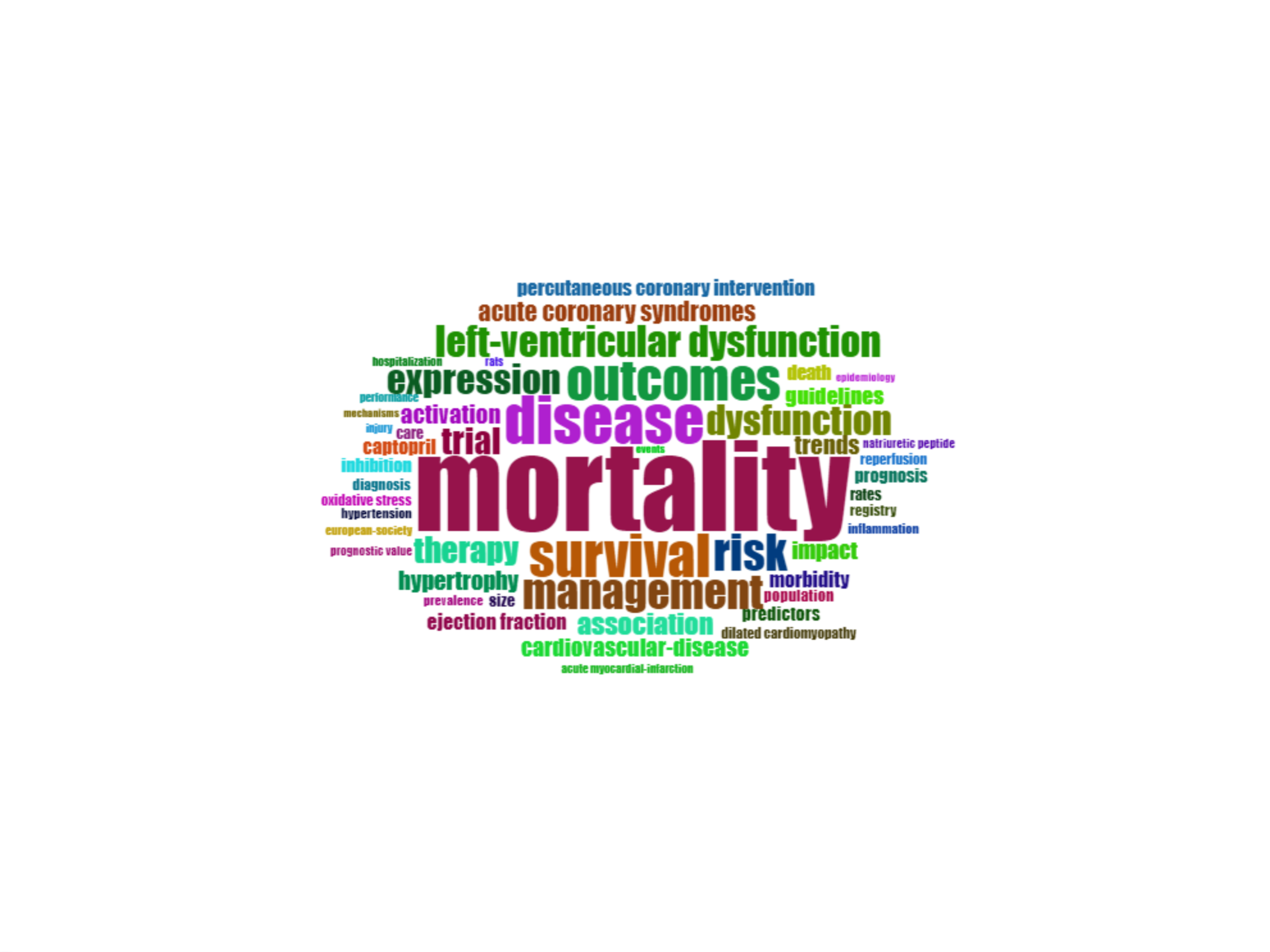

Supplement: Supplementary file 1 [file Datasheet1.zip › figure/7-C.png]

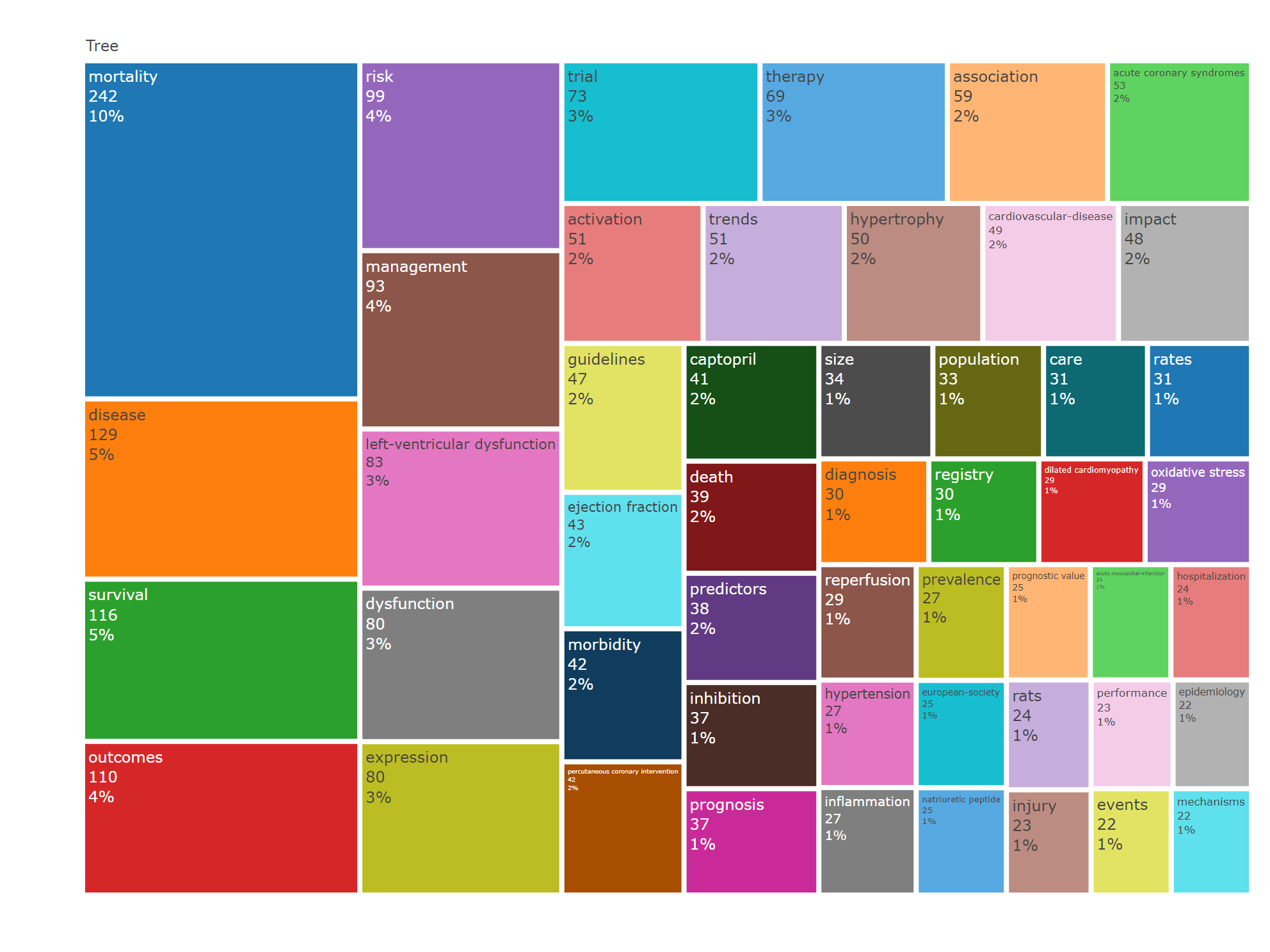

Supplement: Supplementary file 1 [file Datasheet1.zip › figure/7-D.png]

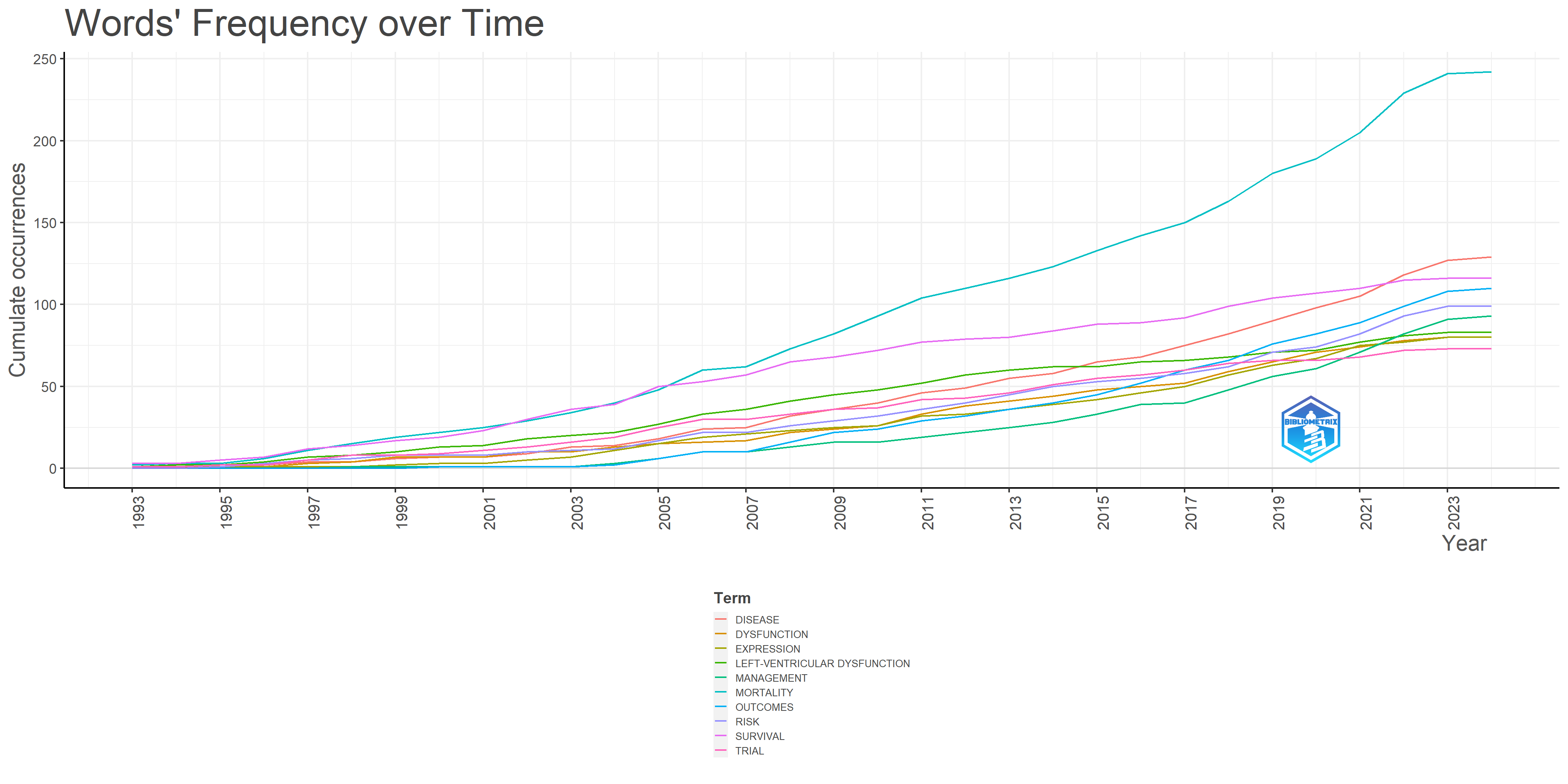

Supplement: Supplementary file 1 [file Datasheet1.zip › figure/7-E.png]

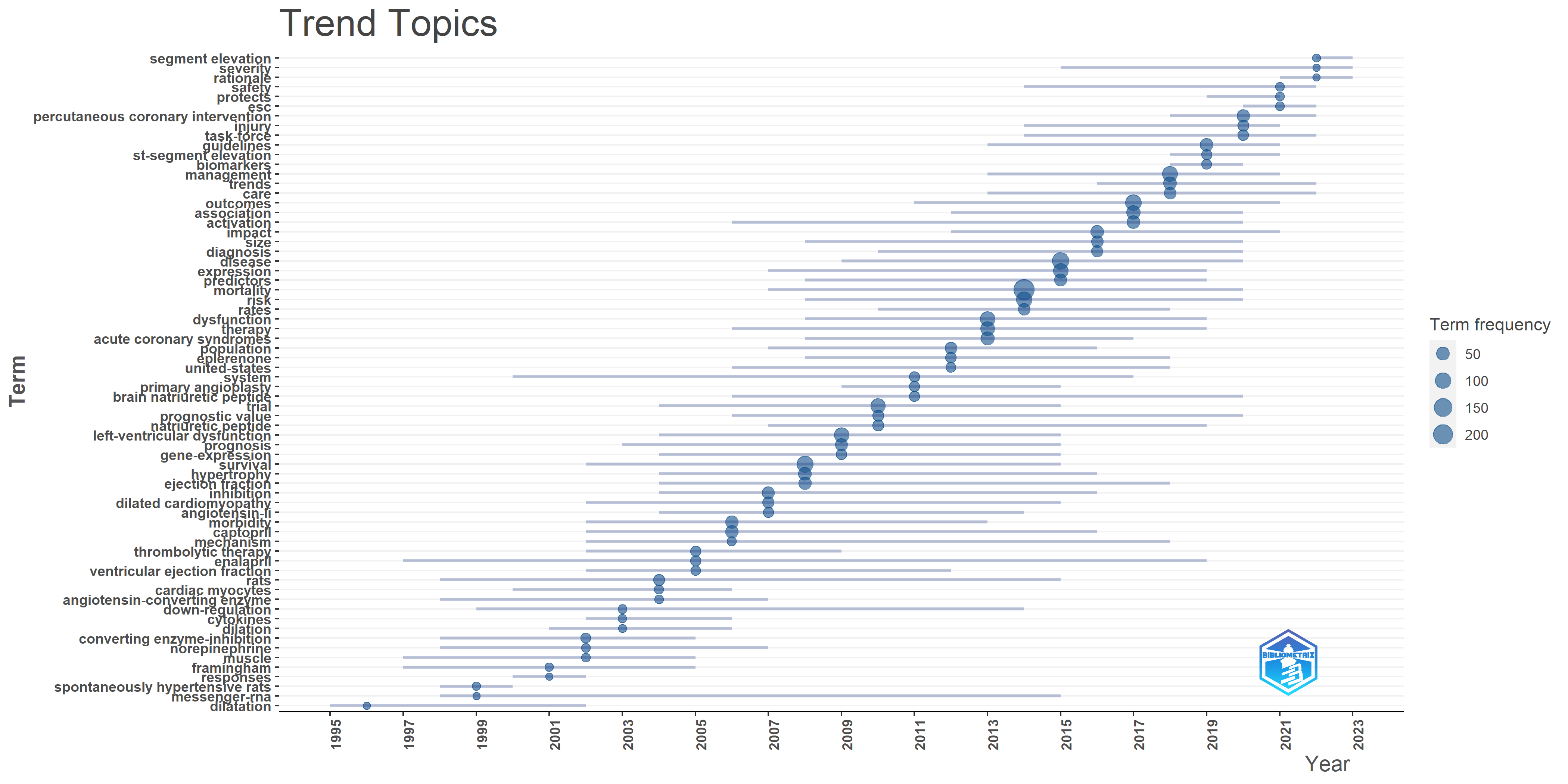

Supplement: Supplementary file 1 [file Datasheet1.zip › figure/7-F.png]

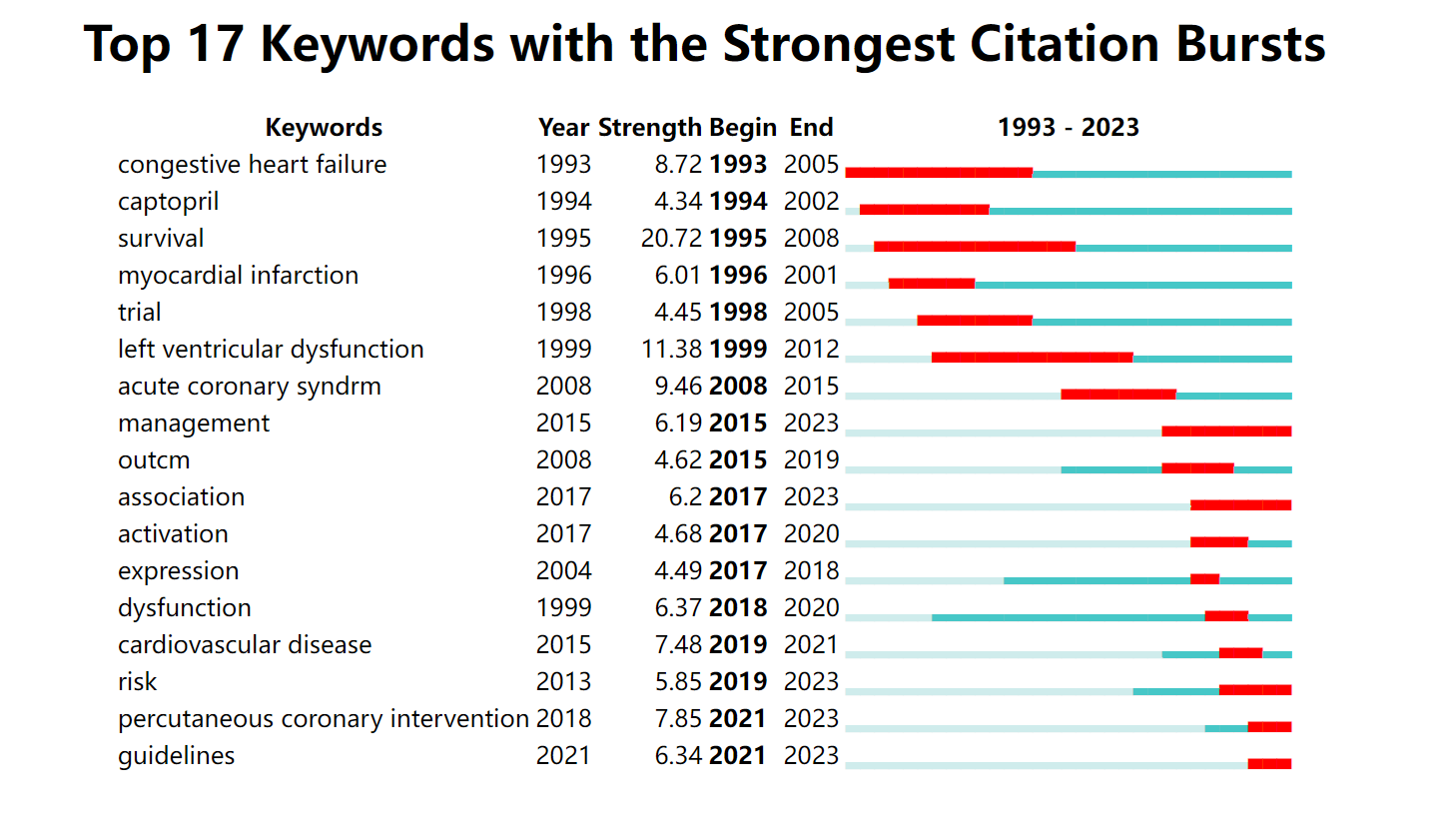

Supplement: Supplementary file 1 [file Datasheet1.zip › figure/8.png]

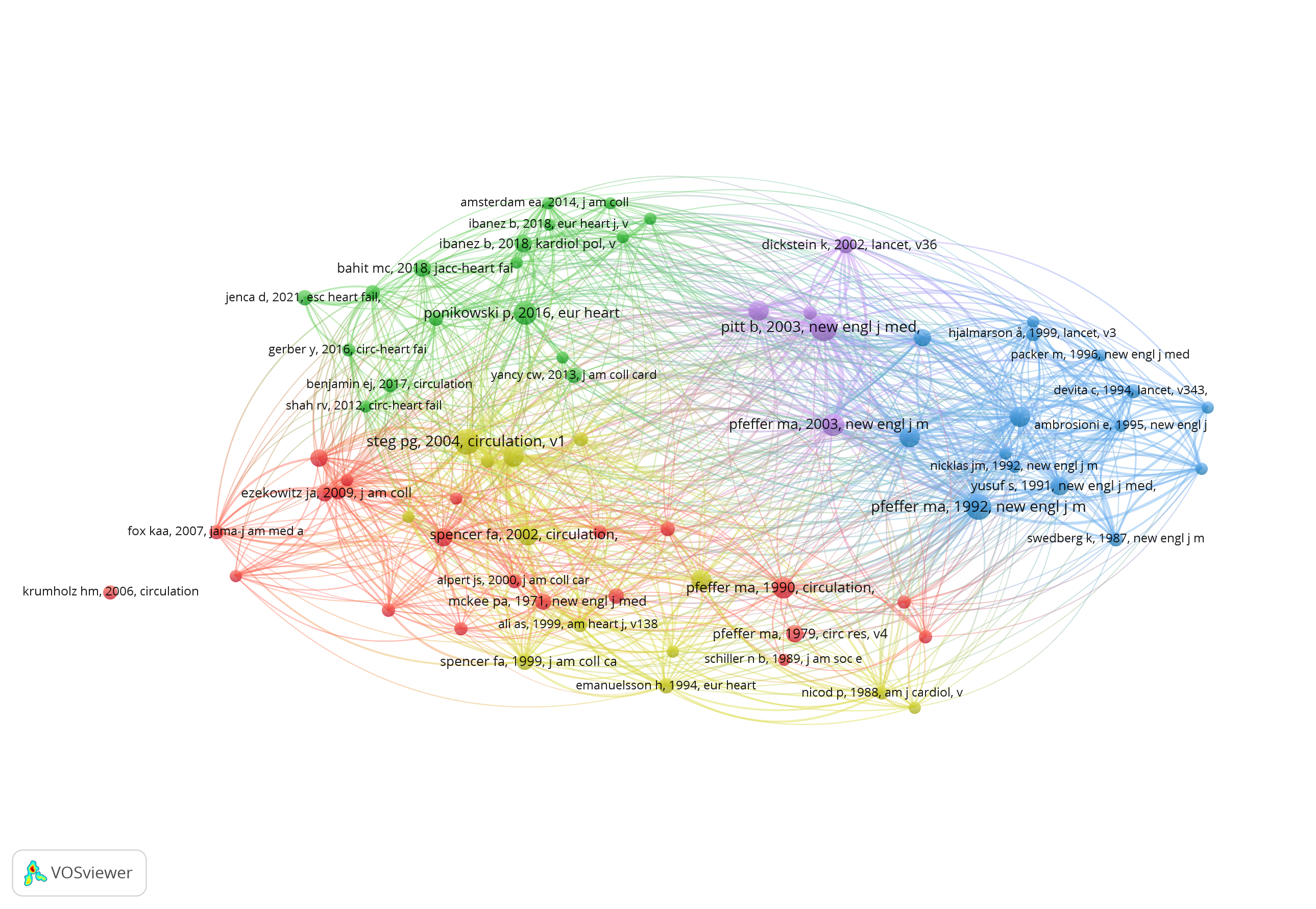

Supplement: Supplementary file 1 [file Datasheet1.zip › figure/9-A.tiff]

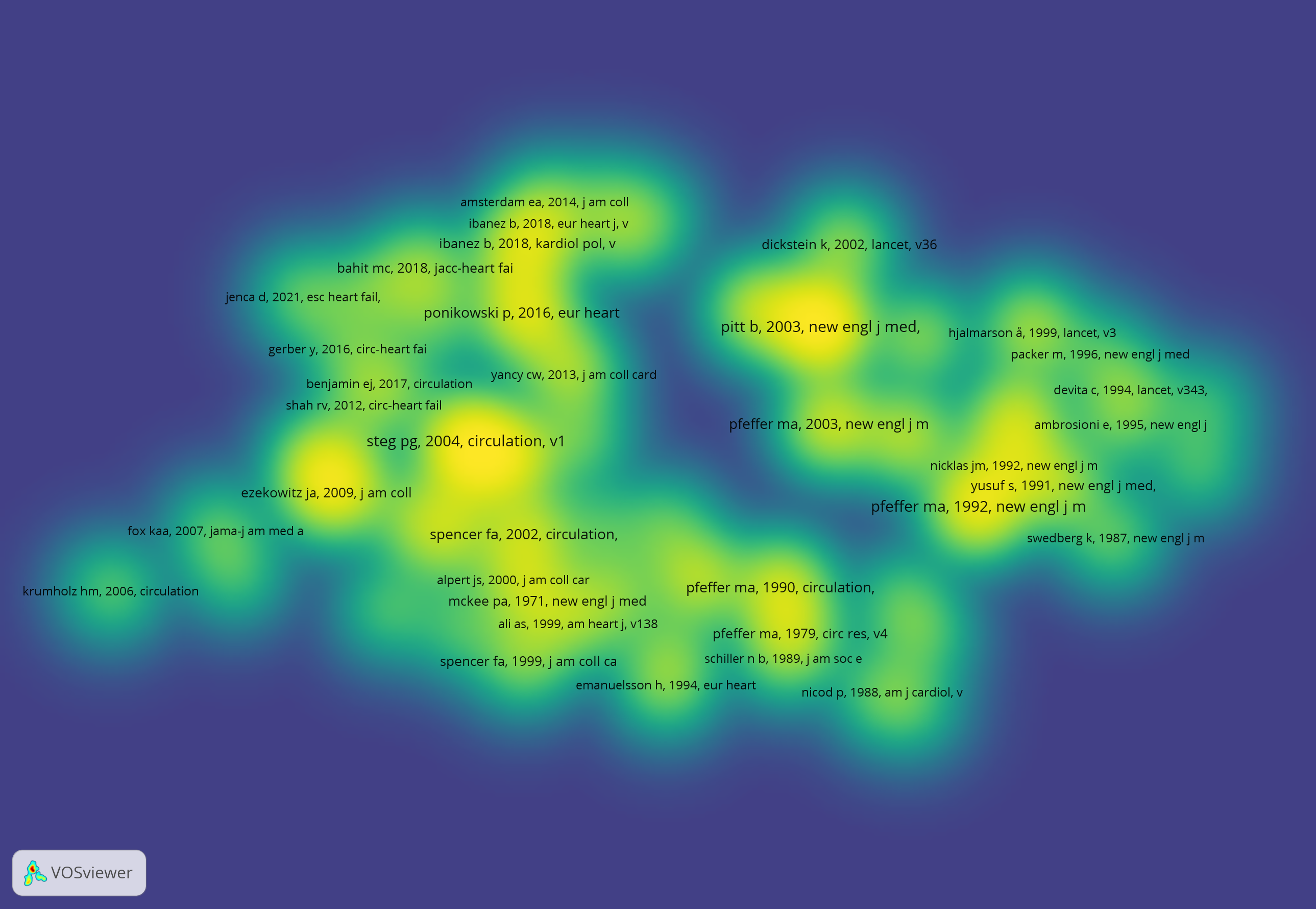

Supplement: Supplementary file 1 [file Datasheet1.zip › figure/9-B.tiff]

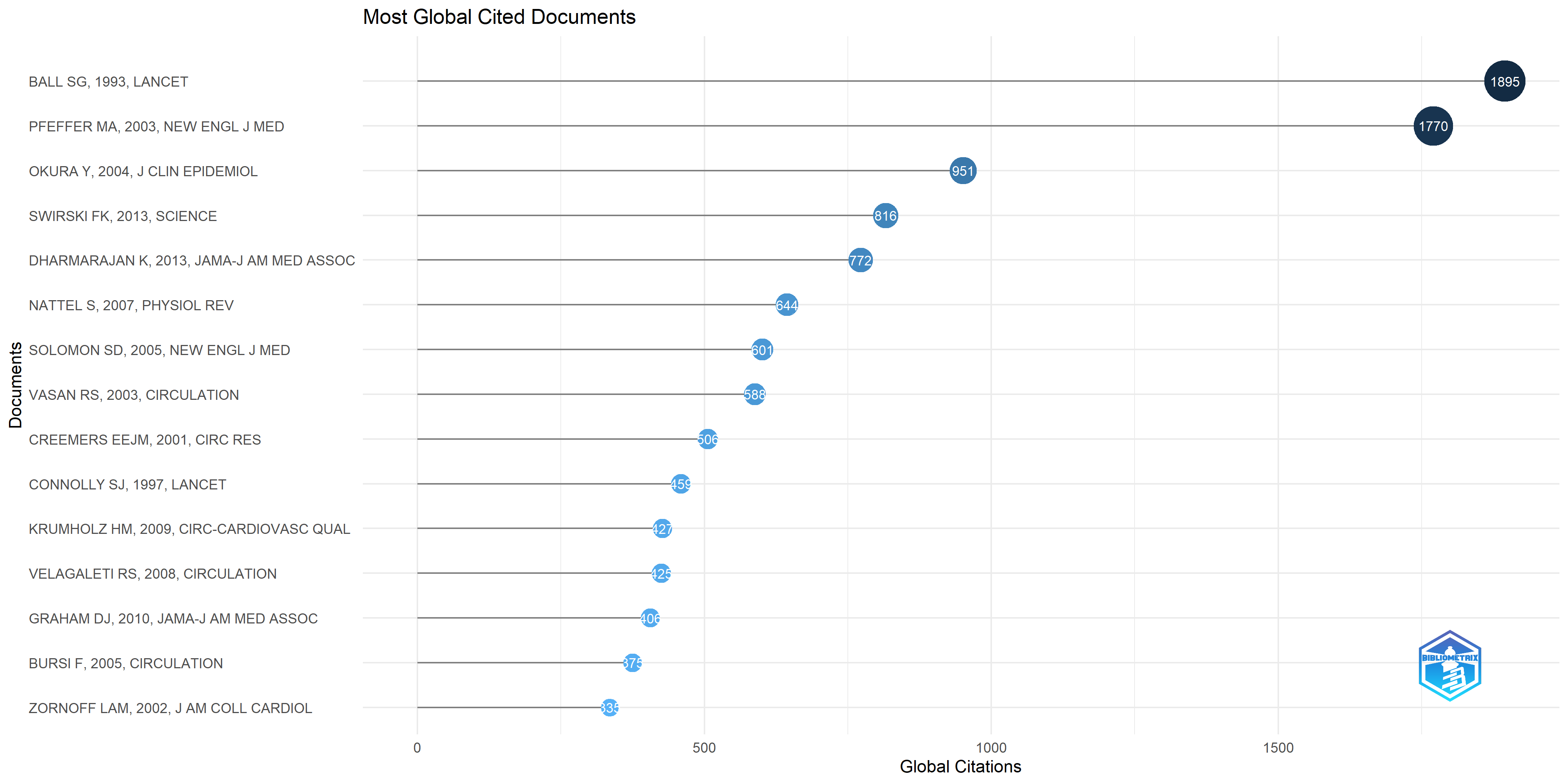

Supplement: Supplementary file 1 [file Datasheet1.zip › figure/9-C.png]
